# Nanopore sequencing reveals *TACC2* locus complexity and diversity of isoforms transcribed from an intronic promoter

**DOI:** 10.1038/s41598-021-88018-9

**Published:** 2021-04-30

**Authors:** Yosuke Ito, Yasuhisa Terao, Shohei Noma, Michihira Tagami, Emiko Yoshida, Yoshihide Hayashizaki, Masayoshi Itoh, Hideya Kawaji

**Affiliations:** 1 Faculty of Medicine, Department of Obstetrics and Gynecology, Juntendo University, 2-1-1 Hongo, Bunkyo, Tokyo, 113-8421 Japan; 2 Preventive Medicine and Applied Genomics Unit, RIKEN Center for Integrative Medical Sciences, 1-7-22 Suehiro-cho, Tsurumi, Yokohama, Kanagawa 230-0045 Japan; 3 Laboratory for Comprehensive Genomic Analysis, RIKEN Center for Integrative Medical Sciences, 1-7-22 Suehiro-cho, Tsurumi, Yokohama, Kanagawa 230-0045 Japan; 4 RIKEN Center for Integrative Medical Sciences, Nucleic Acid Diagnostic System Development Unit, 1-7-22 Suehiro-cho, Tsurumi, Yokohama, Kanagawa 230-0045 Japan; 5 Diagnostics and Therapeutics of Intractable Diseases, Intractable Disease Research Center, Juntendo University Graduate School of Medicine, Tokyo, Japan; 6 RIKEN Preventive Medicine and Diagnosis Innovation Program, 2-1 Hirosawa, Wako, Yokohama, Saitama 351-0198 Japan; 7 Research Center for Genome & Medical Sciences, Tokyo Metropolitan Institute of Medical Science, 2-1-6 Kamikitazawa, Setagaya-ku, Tokyo, 156-8506 Japan; 8 Laboratory for Advanced Genomics Circuit, RIKEN Center for Integrative Medical Sciences, 1-7-22 Suehiro-cho, Tsurumi, Yokohama, Kanagawa 230-0045 Japan

**Keywords:** Molecular biology, Gynaecological cancer

## Abstract

Gene expression is controlled at the transcriptional and post-transcriptional levels. The *TACC2* gene was known to be associated with tumors but the control of its expression is unclear. We have reported that activity of the intronic promoter p10 of *TACC2* in primary lesion of endometrial cancer is indicative of lymph node metastasis among a low-risk patient group. Here, we analyze the intronic promoter derived isoforms in JHUEM-1 endometrial cancer cells, and primary tissues of endometrial cancers and normal endometrium. Full-length cDNA amplicons are produced by long-range PCR and subjected to nanopore sequencing followed by computational error correction. We identify 16 stable, 4 variable, and 9 rare exons including 3 novel exons validated independently. All variable and rare exons reside N-terminally of the TACC domain and contribute to isoform variety. We found 240 isoforms as high-confidence, supported by more than 20 reads. The large number of isoforms produced from one minor promoter indicates the post-transcriptional complexity coupled with transcription at the *TACC2* locus in cancer and normal cells.

## Introduction

Gene expression is tightly regulated at the transcriptional and post-transcriptional levels. Transcription is initiated through recruitment of the transcription pre-initiation complex at a promoter, a proximal upstream region of a transcribed gene^1^. Multiple promoters of a single gene produce distinct transcripts; five or more promoters per gene are reported^2^. The variety of transcripts is further extended by co- or post-transcriptional regulation. In particular, in alternative splicing, different sets of introns are cleaved out from primary transcripts^3^, and three or more isoforms per gene are reported^4^.

Endometrial cancer is the most common type of cancer of the female reproductive tract in developed countries. Only a small fraction of patients has metastasis in lymph nodes within the low-risk patient group, and no established system is available to identify such exceptional cases. We explored the differences of the exceptional cases in transcription through cap analysis of gene expression (CAGE), a method to quantitatively monitor transcription initiation at base-pair resolution^5^. Two genes were found to indicate metastasis in lymph nodes^6^; identification of these molecular markers have opened a way toward preoperative diagnosis. Of them, the gene for transforming acidic coiled-coil-containing protein 2 (*TACC2*)^7^ was found to harbor a novel intronic promoter, p10, whose activity is higher in patients with lymph-node metastasis^6^. The gene encodes a member of the TACC family, which consists of three members that share a conserved C-terminal coiled-coil region, called TACC domain^7,8^. The gene was originally identified as a tumor suppressor in breast cancer^9^, whereas later it was reported as a tumor-promoting factor in prostate cancer^10,11^ and breast cancer^12^. These reports support association of *TACC2* with tumors, but the control of its expression and its effect on tumors remain unclear. Of the eight *TACC2* isoforms reported in 2003^13^, three are around 10-kb long as they have a 4th exon of 5.3 kb, and five isoforms are around 5 kb and do not have this long exon; none of them are initiated from the p10 promoter. Capillary sequencing of cDNA clones derived from p10 revealed that their exons are largely consistent with the ones derived from the canonical promoters^6^. Ten distinct isoforms were found among 16 cDNA clones^6^, indicating the existence of unknown isoforms.

Recent single-molecule long-read sequencing technologies, such as single-molecule real-time (SMRT) sequencing by Pacific Biosciences (PacBio)^14^ and nanopore sequencing by Oxford Nanopore Technologies (ONT)^15,16^, enable us to sequence DNA molecules longer than 10 kb without fragmentation^17,18^. SMRT sequencers measure fluorescence fluctuations that arise from the addition of a specific nucleotide^14^, whereas nanopore sequencers (MinION, GridION, and PromethION) measure ionic current fluctuations that occur when single-stranded nucleic acids pass through nanopores^15^. They make it possible to determine the entire transcript structure without cloning of individual cDNAs. Nanopore sequencing produces longer read lengths with lower sequencing costs, but generates more sequencing errors (about 10%^19,20^) than HiFi reads of SMRT sequencing that relies on consensus among multiple passages of sequencing through circularized template. A protocol to increase sequence accuracy in nanopore sequencers, called 2D sequencing, which determine nucleotides in both strands of DNA molecules sequentially through ligation of a hairpin adapter, was developed once but not available at this moment. Only the method to sequence a single strand, called 1D, is available currently, and there is a demand to obtain accurate sequences with that.

Here, we aimed to provide a comprehensive view of the *TACC2* isoforms derived from the intronic p10 promoter. We used an ONT MinION sequencer to sequence long-range PCR amplicons of the cDNAs, followed by computational error correction. We started by profiling an endometrial cancer cell line (JHUEM-1), and then extended the study to primary tissues of endometrial cancer and normal endometrium. Our results unveiled the diversity of isoforms both in the cell line and primary tissues.

## Material and methods

All experiments on human subjects were conducted in accordance with the declaration of Helsinki.

### JHUEM-1 cell line culture and RNA preparation

The endometrial cancer cell line JHUEM-1 was provided by the RIKEN BRC through the National Bio-Resource Project of the MEXT, Japan. JHUEM-1 cells were cultured in Dulbecco’s modified Eagle’s medium /HamF12 (Sigma-Aldrich Corp., St. Louis, MO, USA) supplemented with 15% fetal bovine serum and 1% penicillin–streptomycin solution. The cells were plated in 6-cm dishes at 10^5^ per dish and incubated for 6 days.

The culture medium was removed by aspiration, the cells were washed with phosphate-buffered saline (PBS), which was removed by aspiration and replaced with PBS containing 0.2% trypsin. Cells were transferred to an RNase-free tube, medium was added, and centrifuged at 300×*g* for 5 min. The supernatant was completely removed by aspiration. Total RNA was extracted with an RNeasy Mini Kit (Qiagen, Valencia, CA, USA) according to the manufacturer’s protocol. Total RNA was diluted to 250 ng/μl based on Nanodrop measurements.

### Patients, sample collection, and RNA preparation

Endometrial cancer tissues and normal endometrium tissues from patients with benign gynecological tumor were obtained from the patients recruited from the Department of Obstetrics and Gynecology, Juntendo University Hospital, Tokyo, Japan, with a written informed consent by following a protocol approved by the ethical review board of Juntendo University Faculty of Medicine. For classification of clinical cases, the patient's information such as histologic subtype, differentiation grade, the International Federation of Gynecology and Obstetrics (FIGO) stage in 2008 and TNM classification were used.

Cancer tissues were cut into 5 mm cubes after hysterectomy, frozen immediately in liquid nitrogen, and stored at − 80 °C. Frozen samples were cut into 30 mg (~ 3 mm cubes) and RNA was extracted with an RNeasy Mini Kit. Total RNA was eluted with 50 µl of RNase-free water. Normal endometrium tissues were cut into 2 mm cubes after hysterectomy, and immediately placed into a PAXgene Tissue FIX Container (Qiagen) in liquid-I (Tissue Fix) at room temperature; after 3 h, they were transferred into liquid-II (Stabilizer) and stored at − 80 °C. RNA was extracted according to the Qiagen protocol for the PAXgene Tissue Container. Total RNA was eluted with 28 µl of RNase-free water. Total RNA was diluted to 250 ng/μl based on Nanodrop measurements.

### Preparation of sequencing target amplicons

cDNA was prepared from total RNA of JHUEM-1 cells and surgical specimens using a PrimeScript II 1st strand cDNA Synthesis Kit (Takara Bio Inc., Kusatsu, Japan). Briefly, samples were denatured at 65 °C for 5 min in the presence of Oligo dT Primer and reverse transcribed at 42 °C for 60 min. The enzyme was inactivated at 70 °C for 15 min, and the samples were immediately chilled at 4 °C. The cDNA was purified with AMPure XP beads (Beckman Coulter, Brea CA, USA), eluted in 50 µl of RNA-free water and 1 µl of the 50 µl was used to prepare *TACC2* p10 amplicons by PCR with LA-taq DNA polymerase and GC Buffer. *TACC2* p10 forward primer (CCAGTTGCTGAAGGGCAGAA) and *TACC2* p10 reverse primer Ex22 (TTGCCTCGAACCTGAGCAATC) were designed from the transcription start site (p10) revealed in a previous study^6^. Thermal cycling was performed for 30 cycles (98 °C for 10 s, 55 °C for 15 s, and 72 °C 60 s). *TACC2* p10 amplicons were purified with AMPure XP beads and eluted with 50 µl of RNA-free water. Their concentration was measured using a Qubit dsDNA BR assay kit (Thermo Fisher Scientific, Waltham, MA, USA) and adjusted to 500 ng per 22.5 µl (22.2 ng/µl). Because the amplicon yield from surgical specimens was low, we performed a second PCR with 1 µl of the 50 µl eluent of *TACC2* p10 PCR amplicon as a template for 15 cycles with the same condition, purified the amplicons and adjusted their concentration as above.

### Nanopore sequencing

Nanopore sequencing libraries were prepared according to the manufacturer’s protocol for 1D Native barcoding genomic DNA using EXP-NBD103 and SQK-LSK108 (ONT, Oxford, UK). The library of JHUEM-1 amplicons was labeled with the NB01–NB03 barcodes, quantified with a Qubit dsDNA HS assay kit (Thermo Fisher Scientific), and 280 ng was used for sequencing. The library of clinical tissue amplicons was labeled with NB01–NB12 barcodes, quantified as above, and 250 ng of amplicons was used for sequencing. Sequencing was performed on MinION MK1b device with MinION Flow Cell R9.4. MinKNOW software version was 1.5.12. Base calling was performed using Albacore ONT Sequencing Workflow Software v2.3.1 with the Basecall Barcoding workflow (ONT).

### Computational processing of MinION read data

We first aligned the reads with the reference genome GRCh37 by using minimap2^21^ with the splice alignment mode option (− x splice). The exon blocks were generated by merging overlapping exons of the primary alignments, and the primary alignments with the same set of exon blocks were grouped. Sequencing errors were corrected with Canu v1.7^22^ by specifying nanopore read (-nanopore-raw) and with additional options corOutCoverage = 999, corMinCoverage = 0, and stopOnReadQuality = false. The corrected reads were subjected to a second round of alignment and grouping. The resulting groups in the second round were expected to agree with individual isoforms more precisely than the ones generated in the first round, as the genome alignments were based on the corrected reads within each of the loosely defined groups in the first round. Within each of the relatively accurate groups generated in the second round, the sequence error correction was conducted again on the original raw reads to avoid bias potentially introduced in the first round owing to incorrect grouping. The script of this error correction step is available as https://github.com/hkawaji/ggec.

The corrected reads were aligned with minimap2 with splice alignment mode (− x splice) and k-mer size 12 (− k 12), and only the primary alignments overlapping with the exons targeted by the amplification primers were kept. Individual exons of the alignments were merged when they overlapped, resulting in 29 non-overlapping merged blocks. The most frequent boundary of the overlapping exons in each block was chosen as the representative exon boundary. Variations of exon boundaries were taken when more than 5% of exon-containing reads were found. Only the alignments matching the representative exon or the identified exon variants were used in isoform counts (Table S4).

### Validation PCR and electrophoresis of target amplicon from JHUEM-1 RNA

cDNA was prepared as described above for the JHUEM-1 cell line, diluted tenfold with RNase-free water, and used as a template for PCR in an ABI 7500 Fast Real-time PCR System (Thermo Fisher Scientific) according to the manufacturer’s instructions. The final concentrations in PCR mixture were as follows: 1× Prime STAR Buffer (Mg^2+^plus), 200 µM dNTP mixture, 0.025 U/µl of PrimeSTAR HS DNA polymerase, 0.52 × SYBR Green (Thermo Fisher Scientific), 0.2 µM forward primer, 0.2 µM reverse primer, and 25 ng/µl cDNA. Thermal cycling was performed for 40 cycles (98 °C for 10 s, 55 °C for 15 s, and 72 °C 60 s). Custom primers for detecting the new exons (Ex2-3, Ex3-4, Ex7-8) were designed by the Primer3 web tool (http://bioinfo.ut.ee/primer3-0.4.0/) and were synthesized by Thermo Fisher Scientific; they are listed in Table S4.

To confirm the amplification with the PCR primers, control synthetic DNA fragments were used (Eurofins Genomics Co., Ltd., Brussels, Belgium). One negative control fragment contained only known *TACC2* exons without novel exons, and three positive control fragments contained novel exons (Fig. S5). The design of the forward primer for rex01 (Ex1-2) and the reverse primer for rex05 (Ex3) has been already published^6^, and these primers were used to amplify the synthetic DNA fragments to assure their quality (Table S5). The new exon custom primers were verified using these control synthetic DNA fragments. The PCR fragments were electrophoresed in 3% NuSieve GTG agarose gel with Tris–borate-EDTA (TBE) buffer for 45 min; 100-bp DNA ladder (Takara Bio, Otsu, Japan) was used as a molecular weight marker. The gel was stained with ethidium bromide to visualize the amplicons. Images were captured using Gel Doc XR plus (Bio-Rad, Hercules, CA, USA).

## Results

### The intronic p10 promoter of *TACC2* is highly active in an endometrial cancer cell line, JHUEM-1

**Figure 1:**
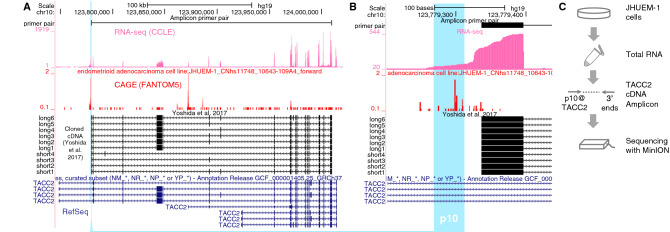
The intronic promoter p10 of the *TACC2* gene is active in the endometrial cancer cell line JHUEM-1. (**A**) Genomic view of the transcriptome (RNA-seq and CAGE) of the *TACC2* locus with known isoforms. Turquoise background indicates the same region of p10, pink and red bars indicate signal intensities of RNA-seq and CAGE. Black and blue boxes indicate exons of gene models previously reported and included RefSeq database. (**B**) Close-up view of the intronic promoter region. (**C**) Schematic representation of our experiments.

Using RNA-seq data from the Cancer Cell Line Encyclopedia (CCLE)^23^^,^^24^, we explored 25 endometrial cancer cell lines with the active p10 promoter of the *TACC2* gene (Table S1) and found that some of them have an RNA-seq signal in the first exon adjacent to the p10 promoter. The expression was most active in JHUEM-1 cells (Fig. S1). The promoter activity was also supported by an unamplified CAGE profile^25^ in the FANTOM5 project^2^ (Fig. 1A,B). We chose the JHUEM-1 cell line for the subsequent analysis.

### Amplicon sequencing of RNA isoforms transcribed from the intronic promoter

To obtain the full-length amplicons of *TACC2* isoforms, we cultured JHUEM-1 cells, extracted RNA, and performed long-range PCR with a primer pair amplifying transcripts from the first exon immediately downstream of p10 to the penultimate exon (Fig. 1C). Using nanopore sequencing, we determined the entire structures of the *TACC2* RNA isoforms transcribed from the p10 promoter.

We found two distinct sizes of amplicons (Fig. S2A), suggesting a subset of the isoforms with the long 4th exon^13^. We performed long-range PCR in triplicate, ligated an adaptor with a barcode sequence unique to each replicate, pooled the replicates, and subjected them to nanopore sequencing. We obtained 869,847 reads in total; their size distribution was consistent with the electrophoresis data (Table S2; Fig. S2).

### Sequence error correction and exon identification

**Figure 2:**
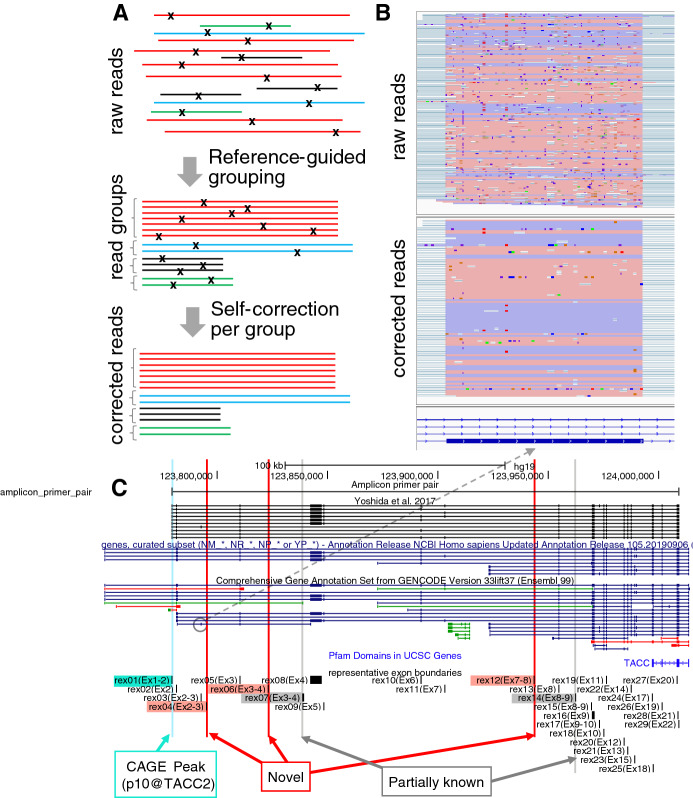
Sequence error correction and identified exons. (**A**) Schematic representation of sequence error correction. Individual lines with the same color indicate the same isoform, where “x” indicates sequence errors (**B**) Sequence matches and mismatches in the alignment before and after sequence error correction, shown in IGV. The pink and blue regions indicate forward and reverse aligned blocks between the reads and the genome. A minor exon, comprising a Gencode gene model ENST00000491540, is shown (referred as rex04). (**C**) The 29 identified exons: rex01 to rex29. Novel exons are colored by red.

Despite the advantage of read length in nanopore sequencing, its drawback is its high error rate (about 10%^19,20^), which makes it challenging to accurately determine exon boundaries through their alignment with the genome. Previous studies using ONT sequencing of long-range PCR amplicons relied on 2D pass reads^26,27^, but we could not use their computational pipelines because our sequencing was based on 1D. Error correction approaches are based on two strategies^28^. The “hybrid” strategy relies on accurate short reads^28^, where additional data has to be obtained separately and correction prioritizes sequences shared among abundant isoforms. This could skew the data toward major isoforms and impede identification of the minor ones. “Self-correction” relies on the other reads obtained from the same experiment^29^, and its direct application to RNA isoforms may still result in skewing the data toward abundant isoforms. We developed an alternative strategy, called “reference-guided self-correction”. The long-read sequences are grouped on the basis of loosely defined exon structures and are subjected to self-correction (Figs. 2A,B, S3). As the correction strategy requires the reads to be aligned with the genome and to belong to a group with multiple members, only a subset (365,177, ~ 40%) of the reads were corrected. We found a fraction of reads derived from other gnomic regions due to non-specific hybridization of the PCR primers, and ones which ends do not necessarily matched to the priming site probably due to incompleteness of error correction. We selected 177,369 (~ 20%) aligned reads that cover the genomic regions corresponding to the primers used for amplification (Table S2). Of them, ~ 4% entirely matched to combinations of 29 representative exon boundaries and their variations defined below.

By aligning the error-corrected reads with the reference genome and examining the aligned blocks, we identified 29 regions and their representative boundaries with the highest frequency per region (Table 1; Figs. 2C, S4). Hereafter, we refer to them using the prefix “rex” for “representative exon regions”, i. e. rex01 to rex29. The outer boundaries of the first and last regions were based on the known gene models, as their internal sequences were the targets of amplification with the PCR primers. Of the 29 regions, 24 exactly matched (at the base-pair level) the exons of the gene models in RefSeq^30^, Ensembl^31^, and a previous report^6^. This confirmation of the known exons indicated the accuracy of our results. We also found three regions, rex04, rex06, and rex12, that did not overlap any known exons, which we considered as candidate novel exons. The remaining two regions were partially overlapping exons of a pseudogene annotated in Ensembl and a transcript model of an EST (expressed sequence tag)-based prediction (SIB Gene in the UCSC Genome Browser Database^32^, https://ccg.epfl.ch/tromer/). We also found length variations in 13 regions (Table 2); in all of them, either the 5′ or 3′ boundaries were consistent with the representative boundaries. The frequencies of exons and isoforms are summarized in Tables S3 and S4.

**Table 1:** Representative exon boundaries identified by long-read sequencing.

Start	End	Name	Length (bp)	Symmetric	Frequency	State	Position in a gene model or reported isoform
123,779,335	123,779,396	rex01 (Ex1-2)	61	No	Stable	Known	Yoshida et al., 1st exon (p10@TACC2 )
123,781,451	123,781,529	rex02 (Ex2)	78	Yes	Stable	Known	Ref seq Gene NM_206862.3 2nd exon
123,792,608	123,792,668	rex03 (Ex2-3)	60	Yes	Rare	Known	Yoshida et al. short variant 4, 3rd exon; Ensemble ENST00000491540, 2nd exon
123,794,962	123,795,075	rex04 (Ex2-3)	113	No	Rare	Novel	NA
123,809,952	123,810,065	rex05 (Ex3)	113	No	Stable	Known	Ref seq Gene NM_206862.3 3rd exon
123,822,912	123,823,005	rex06 (Ex3-4)	93	Yes	Rare	Novel	NA
123,838,491	123,839,151	rex07 (Ex3-4)	660	Yes	Rare	Partially known	Ensembl ENST00000498721, 4th exon
123,842,161	123,847,474	rex08 (Ex4)	5313	Yes	Variable	Known	Ref seq Gene NM_206862.3 4th exon
123,847,992	123,848,106	rex09 (Ex5)	114	Yes	Variable	Known	Ref seq Gene NM_206862.3 5th exon
123,892,123	123,892,249	rex10 (Ex6)	126	Yes	Variable	Known	Ref seq Gene NM_206862.3 6th exon
123,903,086	123,903,221	rex11 (Ex7)	135	Yes	Variable	Known	Ref seq Gene NM_206862.3 7th exon
123,943,555	123,943,720	rex12 (Ex-8)	165	Yes	Rare	Novel	NA
123,954,554	123,954,691	rex13 (Ex8)	137	No	Stable	Known	Ref seq Gene NM_206862.3 8th exon
123,962,081	123,962,141	rex14 (Ex8-9)	60	Yes	Rare	Partially known	SIB Gene, HTR001818.10.1839.19, 8th exon
123,969,388	123,969,484	rex15 (Ex8-9)	96	Yes	Rare	Known	Ref seq Gene NM_001291879.1
123,969,911	123,971,223	rex16 (Ex9)	1312	No	Stable	Known	Ref seq Gene NM_206862.3 9th exon
123,972,856	123,972,892	rex17 (Ex9-10)	36	Yes	Rare	Known	Yoshida et al. long variant 6, 10th exon ; UCSC gene, variat 7, 4th exon
123,974,905	123,974,966	rex18 (Ex10)	61	No	Stable	Known	Ref seq Gene NM_206862.3 10th exon
123,976,141	123,976,343	rex19 (Ex11)	202	No	Stable	Known	Ref seq Gene NM_206862.3 11th exon
123,984,240	123,984,302	rex20 (Ex12)	62	No	Stable	Known	Ref seq Gene NM_206862.3 12th exon
123,985,880	123,985,996	rex21 (Ex13)	116	No	Stable	Known	Ref seq Gene NM_206862.3 13th exon
123,987,351	123,987,523	rex22 (Ex14)	172	NO	Stable	Known	Ref seq Gene NM_206862.3 14th exon
123,988,860	123,989,001	rex23 (Ex15)	141	Yes	Rare	Known	Ref seq Gene NM_206862.3 15th exon
123,996,909	123,997,053	rex24 (Ex17)	144	Yes	Stable	Known	Ref seq Gene NM_206862.3 17th exon
123,997,475	123,997,552	rex25 (Ex18)	77	No	Stable	Known	Ref seq Gene NM_206862.3 18th exon
124,001,472	124,001,516	rex26 (Ex19)	44	No	Stable	Known	Ref seq Gene NM_206862.3 19th exon
124,008,157	124,008,318	rex27 (Ex20)	161	No	Stable	Known	Ref seq Gene NM_206862.3 20th exon
124,008,564	124,008,671	rex28 (Ex21)	107	No	Stable	Known	Ref seq Gene NM_206862.3 21st exon
124,009,058	124,009,089	rex29 (Ex22)	31	No	Stable	Known	Ref seq Gene NM_206862.3 22nd exon

Genomic coordinates (start, end) on chromosome 10 are based on GRCh37 human genome assembly (hg19). Exon names are prefixed by “rex” (e.g., rex01), with exon order in a previous study^6^ indicated in parentheses (e.g., Ex2 indicates the second exon, and Ex2-3 indicates an intron between the second and third exons). When the number of nucleotides in an exon is a multiple of 3, it is referred as symmetric and could be skipped without a change in the reading frame.

**Table 2:** Variations of exon boundaries.

Representative boundaries			Variations		
Name	Length (bp)	Symmetric	Pattern	Length change (BP)	Symmetric
rex07|Ex3-4	660	Yes	L0:R-465	− 465	Yes
rex08|Ex4	5313	Yes	L-4412:R0	− 4412	No
			L-4463:R0	− 4463	No
			L0:R-2445	− 2445	Yes
			L0:R-2656	− 2656	No
			L0:R-4536	− 4536	Yes
			L0:R-5231	− 5231	No
rex09|Ex5	114	Yes	L0:R-19	− 19	No
rex11|Ex7	135	Yes	L-11:R0	− 11	No
			L-47:R0	− 47	No
rex16|Ex9	1312	No	L0:R12	12	Yes
rex17|Ex9-10	36	Yes	L0:R4	4	No
rex18|Ex10	61	No	L-12:R0	− 12	Yes
rex19|Ex11	202	No	L-13:R0	− 13	No
			L0:R93	93	Yes
rex21Ex13	116	No	L-3:R0	− 3	Yes
rex23|Ex15	141	Yes	L-4:R0	− 4	No
			L0:R4	4	No
rex24|Ex17	144	Yes	L9:R0	9	Yes
rex25|Ex18	77	No	L0:R26	26	No
rex26|Ex19	44	No	L0:R4	4	No

Patterns of variations are written as “L value 1:R value 2”, where “L” refers to the 5′-end and “R” indicates changes at the 3′-end. A positive value indicates exon extension, and a negative one indicates exon truncation. For example, L-4412:R0 indicates that the 5′-end is truncated by 4412 bp and the 3′-end is unchanged.

### Validation of the three novel exons in an endometrial cancer cell line

**Figure 3:**
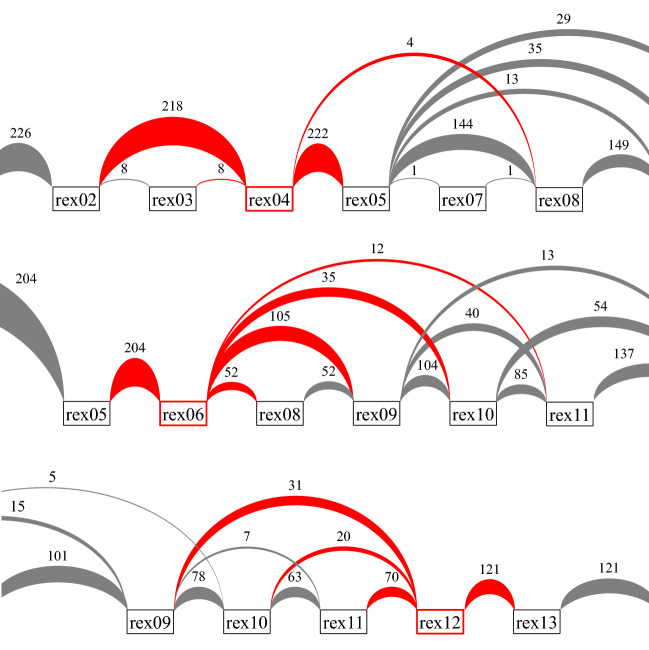
Frequencies of exon–exon junctions of the three novel exons. Black boxes, known exons; red boxes, novel exons. The connections between exons are depicted as arches; frequencies are shown by arch width and numbers.

**Figure 4:**
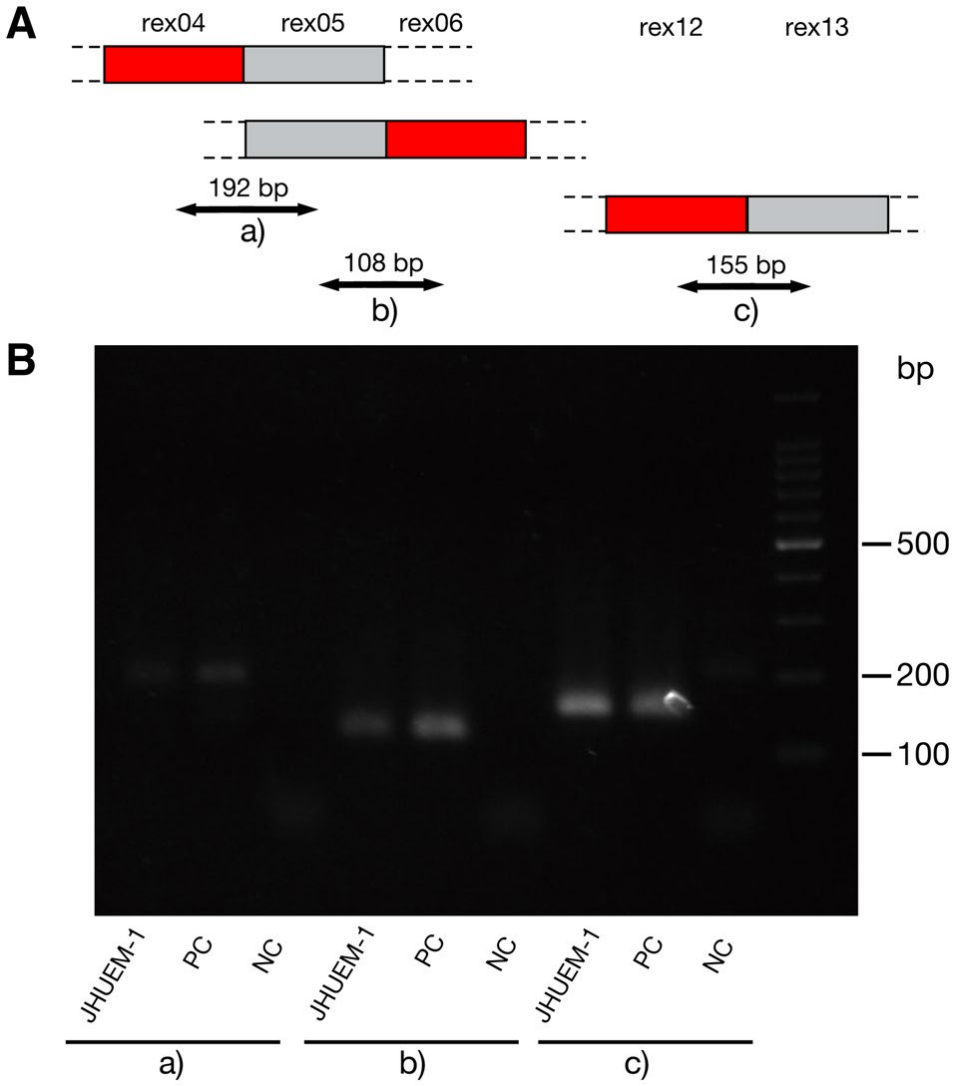
PCR amplification of novel exons. (**A**) Schematic representation of PCR amplicons. (**B**) Electrophoresis of the PCR amplicons. PC and NC indicates synthesized positive and negative controls shown in Table S6. (**a**)–(**c**) indicate the amplicons in (**A**). Full-length image of the gel is included as Fig. S11.

We examined whether the three novel regions represent authentic exons or technical artifacts arising from sequencing errors. In an independent experiment, we conducted PCR analysis with primers specific to the sequences of the novel regions (Fig. 3). The designed primers targeted the most frequent exon–exon junctions (Figs. 4A, S5, and Table S5). cDNAs prepared from JHUEM-1 cells, and synthetic positive and negative controls were subjected to PCR (Table S6). A single band of the expected size was found in the JHUEM-1 cell sample and in the positive but not negative control for each amplicon (Fig. 4B). These results confirmed the three regions as authentic exons.

### Long-read sequencing of RNA isoforms in primary tissues

Next, we analyzed 16 cases of endometrial cancer and 5 normal endometria (Table S7), as transcripts from the intronic promoter p10 of *TACC2* were detectable in normal endometria at a similar level to lower cases of primary tumors. We used the same approach as above, but performed two PCR rounds to obtain enough material for sequencing because the RNA amount was limited. We also found two distinct sizes of amplicons (Fig. S6), but the bands corresponding to the larger isoforms were sometimes faint. This is likely due to less efficient amplification of long molecules with the additional round of PCR. We obtained two pools of full-length cDNA amplicons; each of them consisted of 12 samples with unique sequence adaptors (Table S7). Sequencing of the amplicons produced almost ~ 5.5 million reads in total (~ 231 thousand reads on average per sample). We corrected sequencing errors as above and obtained ~ 1.7 million corrected reads (~ 72 thousand on average per sample; ~ 30%; Table S7). The ratio was lower than for JHUEM-1 cells (~ 40%) but the number of corrected reads was larger than in the individual JHUEM-1 replicates. Approximately 179 thousand reads (~ 8 thousand on average per sample; ~ 3%) matched exactly the exons defined above. Although we obtained the full-length sequences of *TACC2* from the primary tissues, quantification of isoform abundance was limited because of the additional round of long-range PCR.

### Diversity of isoform structures

Our full-length sequence data contained 27 profiles, comprising the triplicates for the cell line and 21 primary tissues, including three duplicates for primary tumors. Approximately 200 thousand full-length error-corrected sequences (8 thousand sequences per profile on average; Tables S2 and S7) were obtained, with each genome alignment exactly matching the 29 representative exons or their 21 variations.

We confirmed 9 of the 10 published isoforms^6^; 6 isoforms were within the top 10 by the number of reads. The six ones were supported with more than 6000 reads, 2 within the top 50 with more than 500 reads, and one was at the 240th place with 20 reads (Tables S4 and S8. The only missing isoform was expected to have a 60-bp exon, where the exon itself (rex03) was found in our data set with less than 1% frequency (Table 1).

We next assessed the reproducibility of isoform frequencies based on the read counts of technical replicates. In JHUEM-1 cells (Fig. S7), technical replicates rep1 and rep2 were more consistent with each other (Spearman correlation coefficient (*r*_*s*_) = 0.88) than with rep3 (*r*_*s*_ = 0.78). On the other hand, in primary tumors, correlation was poor (*r*_*s*_ < 0.5 in the examples shown in Fig. S8). Principle component analysis of isoform frequencies (Fig. S9) did not show clear segregation of sample types, while one could assume cell or tissue specific patterns in an analogous way to tissue-specific alternative splicing^3^. The accuracy of isoform quantification was good for the cell line but limited for primary tissues, probably because of an additional amplification step of long-range PCR, and it is not practical to compare isoform frequencies among different samples by using the present data.

**Figure 5:**
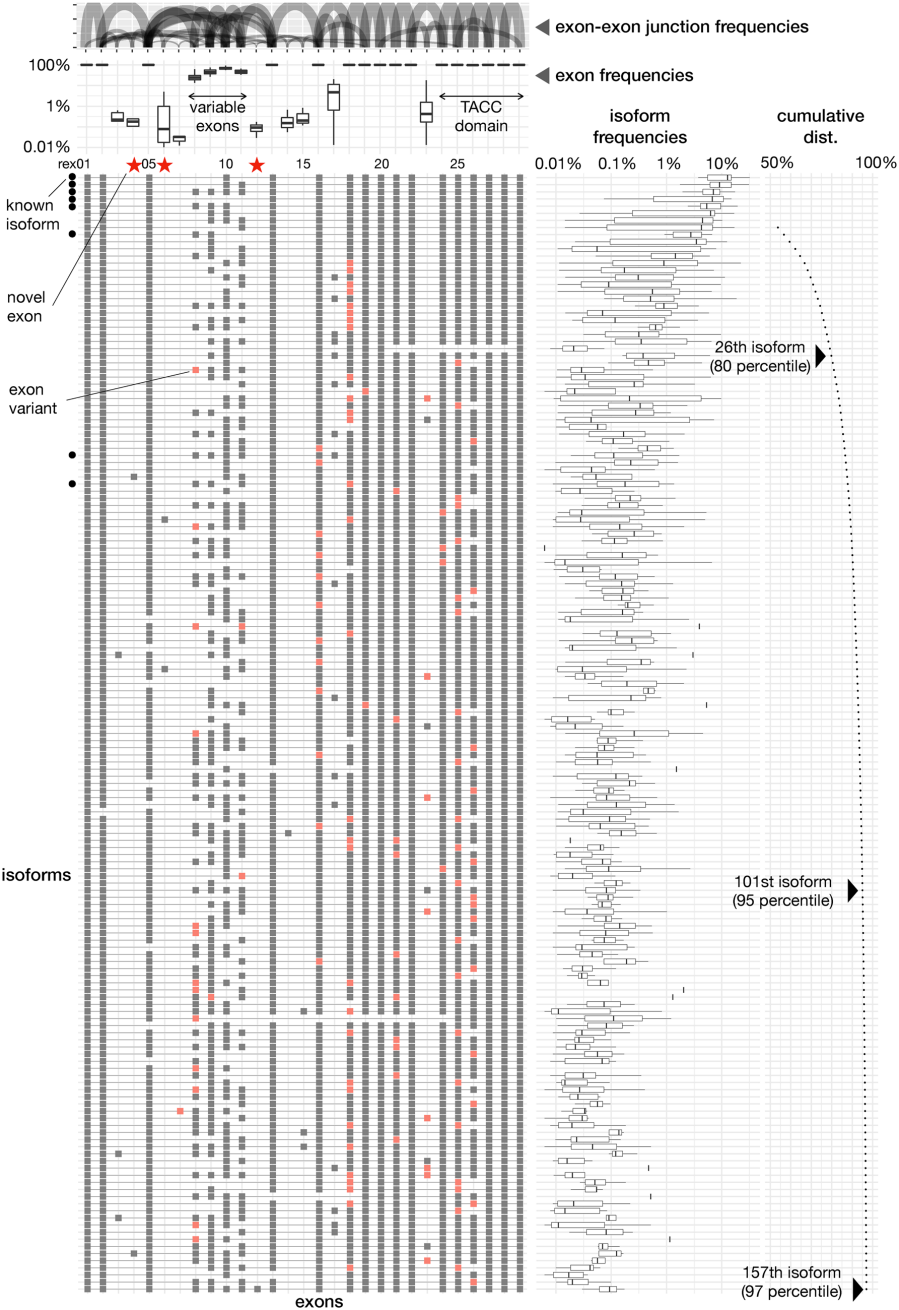
Isoforms transcribed from the TACC2 intronic promoter. Exon structures of 157 isoforms, where the validated novel exon rex12 firstly appears in 157th, are indicated by gray or pink boxes in the main (left bottom) panel. Isoform frequencies are shown by box plots in the middle panel, where frequency is calculated through dividing the number of corrected reads corresponding to the isoform by the total corrected reads per profile. The isoforms are ordered from the top by their total of corrected reads, the number of supporting evidences. Cumulative distribution is shown in the right panel.

Therefore, we focused on qualitative features rather than quantification. The overall frequencies of isoforms, exons, and exon–exon junctions, and isoform structures are shown in Fig. 5. The isoforms were ranked by frequency, as it provides supporting evidence of the isoform presence. The most frequent one corresponded to only 10% of the reads, suggesting that there are no dominant isoforms; 26 isoforms accounted for 80% of the reads and 101 isoforms accounted for 95%. Notably, the top isoforms show high frequencies in the profiled samples, while the quantification accuracy is limited. To cover all known and newly discovered exons, isoforms down to the 157th isoform were required (Fig. 5). This is consistent with the observation mentioned above that the 240th isoform matched a known isoform.

The exon frequencies indicated the presence of three exon classes: 16 stable exons present in nearly all isoforms, 4 variable exons present in more than 10% of the reads, and 9 rare exons present in as few as 0.01–10% of the reads. The three novel exons belonged to the class of rare exons. The functionally characterized TACC domain is located in the C-terminal part of the protein, and all exons encoding this domain were stably included. We found that the variations arise from the N-terminal part, which is similar to that in *TACC2* isoforms transcribed from the canonical upstream promoters. The top 12 isoforms arose only from the combinations of variable exons, while a variant of the 6th residential exon lacking 12 bp from its start position appeared repeatedly in the subsequent isoforms (Fig. S10).

## Discussion

Here we used nanopore sequencing to extensively profile *TACC2* isoforms derived from the intronic promoter p10. We chose nanopore sequencing because of low installation costs, simple operation, and use in other studies in which 2D sequencing was performed^26,27^ (although we used 1D sequencing). Because this approach has a higher error rate than 2D sequencing or circular consensus sequencing of PacBio (an improved version of SMRT sequencing), we developed a strategy called “reference-guided self-correction” to correct errors in each isoform-level group. This strategy substantially increased base accuracy and reduced the number of available reads to only 3% to 4% of the original reads. Unique molecular identifier (UMI), random oligonucleotide sequences uniquely attached to individual PCR templates, is successfully applied to increase sequence accuracy in a recent study^33^. It performs error correction within the reads obtained from the same PCR template while our strategy performs the correction within the same isoform. The two strategies approaching the same problem with different levels can be used complementary. UMI-based approach could be applied to a larger potion of the reads as it does not require the reads to be mapped on the genome, and remaining reads with insufficient error-correction at molecular level could be corrected at isoform level. Further, our strategy of isoform-level error correction would be useful for PCR-free applications, such as RNA direct sequencing.

We confirmed two major sizes of *TACC2* isoforms, ~ 4 kb and 10 kb (Fig. S2), all previously reported exons^6^ (Fig. 2; Table 1), and 9 of 10 reported isoforms^6^ (Fig. 5). Precise matches of the identified exon boundaries to the reported ones emphasize the accuracy of our results at the base-pair level. The absence of the 16th exon in one of the isoforms derived from the most upstream promoter was also consistently found in the previous study^6^ and our data. The 15th exon was absent in 10 isoforms in the previous study^6^, and its frequency was lower than 1% in our data set (referred to as rex23; Fig. 5, Table S3). The isoform missing from our data includes a rare exon (rex03, < 1% frequency). It is likely to be absent in the samples we studied, or to be present below the detection limit. We further uncovered three novel exons, which was validated with PCR independently to the sequencing. Overall, we identified 29 exons, and all of them are supported by previous reports, existing databases, and our own confirmatory experiments.

Our data demonstrate strong preference of exon usage: 16 stable, 4 variable, and 9 rare exons. The longest exon (rex08) is variable; it contributed to both bands on the gel electrophoresis of full-length cDNAs. Exons rex09, rex10, and rex11 are also variable. If the four variable exons were chosen randomly, 16 (= 2^4^) isoforms could be present with high frequencies, but only 12 isoforms were present with top frequencies, indicating unrevealed regulation of exon selection. These four consecutive exons span almost 50 kbp on the genome (Fig. 2), and repressive regulation at the post-transcriptional level rather than co-transcriptional regulation seems more likely.

Rare exons also contributed to isoform diversity. Eight of 9 (88%) rare exons and all 4 variable exons were symmetric (i.e., their nucleotide numbers were multiples of 3), whereas only 2 of 16 (12%) stable exons were symmetric (Table 2). This is consistent with the reported preference of alternative exons^34^. Notably, the regions encoded by the variable and rare exons were located N-terminally of the functionally characterized and cross-species conserved region, TACC domain^35^. As they do not disrupt the coding frame, the C-terminal domain is preserved in most of the isoforms.

With the 29 identified exons and their 21 variations, we found 1,464 isoforms, including those observed only once, and 240 isoforms were supported by at least 20 reads. The 240th isoform has been independently identified by cDNA cloning with capillary sequencing^6^. We consider those observed at least 20 times as high-confidence isoforms. If we set the threshold at 3 times per isoform, we found 694 isoforms. Our data unveiled 240 isoforms with high confidence and additional 454 with minimum evidence. Isoform diversity in conjunction with the frequency of exons and exon–exon junctions is shown in Fig. 5.

Diverse isoforms were identified for *BRCA1*^27^ and *CACNA1C*^26^. In the case of *BRCA1* having ~ 24 exons, 32 isoforms were identified, including 18 novel ones found in the control lymphoblastoid cells but not in breast cancer tissues. These include aberrant isoforms that are usually degraded by nonsense-mediated RNA decay, upon inhibition of the decay by culturing cells with cycloheximide. The latter study^26^ identified 241 isoforms and 38 novel exons in the *CACNA1C* gene, where known isoforms consist of ~ 50 exons. The large number of exons facilitates the great diversity of isoforms. In the *TACC2* gene, we found 240 isoforms with high-confidence and 694 isoforms with minimum evidence, based on 29 exons including 3 novel ones. Our data demonstrates that isoform diversity can be increased even with a limited number of exons.

Not all the data on the association of *TACC2* expression with tumors are consistent. Chen et al.^9^ reported it as a tumor suppressor in breast cancer, but Cheng et al.^12^ reported its oncogenic effect in the same type of cancer. Takayama et al.^10,11^ suggested its positive effect on tumor growth in prostate cancer. While we did not find any relationships between isoform abundance and cell types, probably because of technical limitations of this study, the discordant reports could be caused by the complexity of gene structure and isoform diversity. Concordant results of tumor associations could be obtained through careful discrimination of these isoforms.

## Supplementary Information

Supplementary Information 1.

Supplementary Information 2.

## Data Availability

The results of nanopore sequencing of JHUEM-1 cells are available at DDBJ DRA under accession DRA011097 (https://ddbj.nig.ac.jp/DRASearch/submission?acc=DRA011097).

## References

[1] Roeder RG (1996). The role of general initiation factors in transcription by RNA polymerase II. Trends Biochem. Sci..

[2] Consortium F (2014). A promoter-level mammalian expression atlas. Nature.

[3] Baralle FE, Giudice J (2017). Alternative splicing as a regulator of development and tissue identity. Nat. Rev. Mol. Cell Biol..

[4] Harrow J, Denoeud F, Frankish A, Reymond A, Chen CK, Chrast J, Lagarde J, Gilbert JG, Storey R, Swarbreck D, Rossier C (2006). GENCODE: producing a reference annotation for ENCODE. Genome Biol..

[5] Murata M (2014). Detecting expressed genes using CAGE. Methods Mol. Biol..

[6] Yoshida E (2017). Promoter-level transcriptome in primary lesions of endometrial cancer identified biomarkers associated with lymph node metastasis. Sci. Rep..

[7] Still IH, Hamilton M, Vince P, Wolfman A, Cowell JK (1999). Cloning of TACC1, an embryonically expressed, potentially transforming coiled coil containing gene, from the 8p11 breast cancer amplicon. Oncogene.

[8] Gergely F, Kidd D, Jeffers K, Wakefield JG, Raff JW (2000). D-TACC: a novel centrosomal protein required for normal spindle function in the early Drosophila embryo. EMBO J..

[9] Chen H-M (2000). AZU-1: a candidate breast tumor suppressor and biomarker for tumor progression. Mol. Biol. Cell.

[10] Takayama K (2012). TACC2 is an androgen-responsive cell cycle regulator promoting androgen-mediated and castration-resistant growth of prostate cancer. Mol. Endocrinol..

[11] Takayama K, Inoue S (2013). Transcriptional network of androgen receptor in prostate cancer progression. Int. J. Urol..

[12] Cheng S, Douglas-Jones A, Yang X, Mansel RE, Jiang WG (2010). Transforming acidic coiled-coil-containing protein 2 (TACC2) in human breast cancer, expression pattern and clinical/prognostic relevance. Cancer Genom. Proteom..

[13] Lauffart B, Gangisetty O, Still IH (2003). Molecular cloning, genomic structure and interactions of the putative breast tumor suppressor TACC2. Genomics.

[14] Rhoads A, Au KF (2015). PacBio sequencing and its applications. Genom. Proteom. Bioinform..

[15] Jain M, Olsen HE, Paten B, Akeson M (2016). The Oxford Nanopore MinION: delivery of nanopore sequencing to the genomics community. Genome Biol..

[16] Jain M (2018). Nanopore sequencing and assembly of a human genome with ultra-long reads. Nat. Biotechnol..

[17] Treutlein B, Gokce O, Quake SR, Sudhof TC (2014). Cartography of neurexin alternative splicing mapped by single-molecule long-read mRNA sequencing. Proc. Natl. Acad. Sci. U.S.A..

[18] Norris AL, Workman RE, Fan Y, Eshleman JR, Timp W (2016). Nanopore sequencing detects structural variants in cancer. Cancer Biol. Ther..

[19] Cherf GM (2012). Automated forward and reverse ratcheting of DNA in a nanopore at 5-A precision. Nat. Biotechnol..

[20] Koren S (2012). Hybrid error correction and de novo assembly of single-molecule sequencing reads. Nat. Biotechnol..

[21] Li H (2018). Minimap2: pairwise alignment for nucleotide sequences. Bioinformatics.

[22] Koren S (2017). Canu: scalable and accurate long-read assembly via adaptive k-mer weighting and repeat separation. Genome Res..

[23] Barretina J (2012). The cancer cell line encyclopedia enables predictive modelling of anticancer drug sensitivity. Nature.

[24] Cancer Cell Line Encyclopedia C, Genomics of Drug Sensitivity in Cancer C (2015). Pharmacogenomic agreement between two cancer cell line data sets. Nature.

[25] Kanamori-Katayama M (2011). Unamplified cap analysis of gene expression on a single-molecule sequencer. Genome Res..

[26] Clark MB (2020). Long-read sequencing reveals the complex splicing profile of the psychiatric risk gene CACNA1C in human brain. Mol. Psychiatry.

[27] de Jong LC (2017). Nanopore sequencing of full-length BRCA1 mRNA transcripts reveals co-occurrence of known exon skipping events. Breast Cancer Res..

[28] Zhao L (2019). Analysis of transcriptome and epitranscriptome in plants using PacBio Iso-Seq and nanopore-based direct RNA sequencing. Front. Genet..

[29] Salmela L, Walve R, Rivals E, Ukkonen E (2017). Accurate self-correction of errors in long reads using de Bruijn graphs. Bioinformatics.

[30] Rajput B, Pruitt KD, Murphy TD (2019). RefSeq curation and annotation of stop codon recoding in vertebrates. Nucleic Acids Res..

[31] Yates AD (2020). Ensembl 2020. Nucleic Acids Res..

[32] Lee CM (2020). UCSC genome browser enters 20th year. Nucleic Acids Res..

[33] Karst SM (2021). High-accuracy long-read amplicon sequences using unique molecular identifiers with Nanopore or PacBio sequencing. Nat. Methods.

[34] Magen A, Ast G (2005). The importance of being divisible by three in alternative splicing. Nucleic Acids Res..

[35] Padhi BK, Zigler JS, Padhi P, Hose S, Sinha D (2014). Expression pattern of an evolutionarily conserved splice variant in the rat Tacc2 gene. Genesis.

