# Publisher Correction: Nanopore sequencing reveals *TACC2* locus complexity and diversity of isoforms transcribed from an intronic promoter

**DOI:** 10.1038/s41598-021-96196-9

**Published:** 2021-08-13

**Authors:** Yosuke Ito, Yasuhisa Terao, Shohei Noma, Michihira Tagami, Emiko Yoshida, Yoshihide Hayashizaki, Masayoshi Itoh, Hideya Kawaji

**Affiliations:** 1 Faculty of Medicine, Department of Obstetrics and Gynecology, Juntendo University, 2-1-1 Hongo, Bunkyo, Tokyo, 113-8421 Japan; 2 Preventive Medicine and Applied Genomics Unit, RIKEN Center for Integrative Medical Sciences, 1-7-22 Suehiro-cho, Tsurumi, Yokohama, Kanagawa 230-0045 Japan; 3 Laboratory for Comprehensive Genomic Analysis, RIKEN Center for Integrative Medical Sciences, 1-7-22 Suehiro-cho, Tsurumi, Yokohama, Kanagawa 230-0045 Japan; 4 RIKEN Center for Integrative Medical Sciences, Nucleic Acid Diagnostic System Development Unit, 1-7-22 Suehiro-cho, Tsurumi, Yokohama, Kanagawa 230-0045 Japan; 5 Diagnostics and Therapeutics of Intractable Diseases, Intractable Disease Research Center, Juntendo University Graduate School of Medicine, Tokyo, Japan; 6 RIKEN Preventive Medicine and Diagnosis Innovation Program, 2-1 Hirosawa, Wako, Yokohama, Saitama 351-0198 Japan; 7 Research Center for Genome & Medical Sciences, Tokyo Metropolitan Institute of Medical Science, 2-1-6 Kamikitazawa, Setagaya-ku, Tokyo, 156-8506 Japan; 8 Laboratory for Advanced Genomics Circuit, RIKEN Center for Integrative Medical Sciences, 1-7-22 Suehiro-cho, Tsurumi, Yokohama, Kanagawa 230-0045 Japan

Correction to: *Scientific Reports* https://doi.org/10.1038/s41598-021-88018-9, published online 30 April 2021

The original version of this Article contained errors in the Affiliations.

Affiliation 8 was a duplicate of affiliation 5. The correct affiliation 8 is listed below.

Laboratory for Advanced Genomics Circuit, RIKEN Center for Integrative Medical Sciences, 1-7-22 Suehiro-cho, Tsurumi, Yokohama, Kanagawa 230-0045, Japan.

Consequently, affiliation 8 was removed from authors Yosuke Ito and Hideya Kawaji.

In addition, Masayoshi Itoh was incorrectly affiliated with ‘Laboratory for Comprehensive Genomic Analysis, RIKEN Center for Integrative Medical Sciences, 1-7-22 Suehiro-cho, Tsurumi, Yokohama, Kanagawa 230-0045, Japan.’

The correct affiliations are listed below:

RIKEN Preventive Medicine and Diagnosis Innovation Program, 2-1 Hirosawa, Wako, Yokohama, Saitama 351-0198, Japan.

Laboratory for Advanced Genomics Circuit, RIKEN Center for Integrative Medical Sciences, 1-7-22 Suehiro-cho, Tsurumi, Yokohama, Kanagawa 230-0045, Japan.

The original Article and accompanying Supplementary Information files have been corrected.

