# Supplementary material for: Nanopore sequencing reveals TACC2 locus complexity and diversity of isoforms transcribed from an intronic promoter
Source: Sci Rep. 2021 Apr 30;11:9355. doi: 10.1038/s41598-021-88018-9 (PMC8087818; doi:10.1038/s41598-021-88018-9)
Supplement: Supplementary file 1 — Supplementary Information 1. [file 41598_2021_88018_MOESM1_ESM.pptx]

## Slide 1
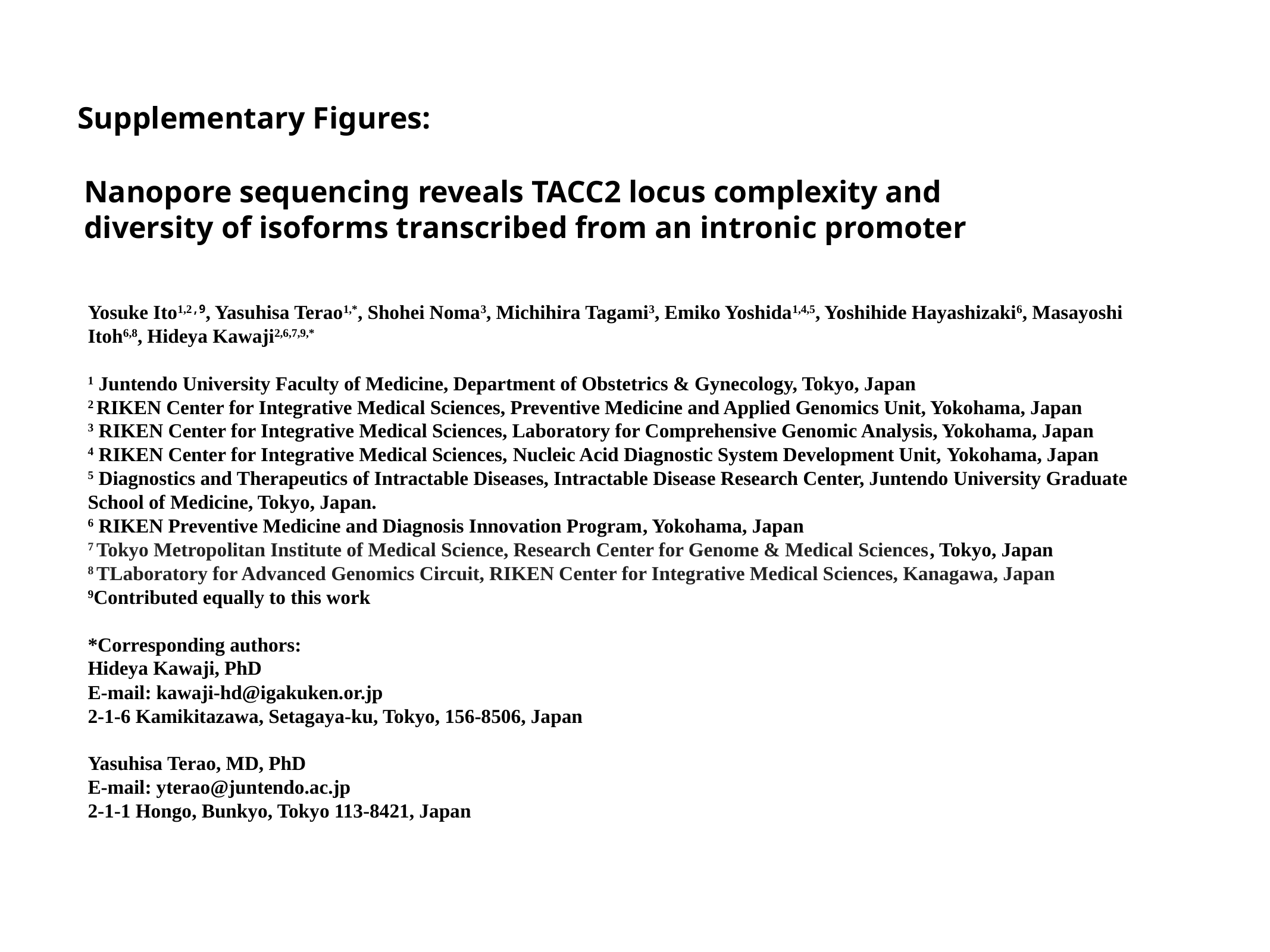

Supplementary Figures:
Nanopore sequencing reveals TACC2 locus complexity and diversity of isoforms transcribed from an intronic promoter
Yosuke Ito1,2,9, Yasuhisa Terao1,*, Shohei Noma3, Michihira Tagami3, Emiko Yoshida1,4,5, Yoshihide Hayashizaki6, Masayoshi Itoh6,8, Hideya Kawaji2,6,7,9,*
1 Juntendo University Faculty of Medicine, Department of Obstetrics & Gynecology, Tokyo, Japan
2 RIKEN Center for Integrative Medical Sciences, Preventive Medicine and Applied Genomics Unit, Yokohama, Japan
3 RIKEN Center for Integrative Medical Sciences, Laboratory for Comprehensive Genomic Analysis, Yokohama, Japan
4 RIKEN Center for Integrative Medical Sciences, Nucleic Acid Diagnostic System Development Unit, Yokohama, Japan
5 Diagnostics and Therapeutics of Intractable Diseases, Intractable Disease Research Center, Juntendo University Graduate School of Medicine, Tokyo, Japan.
6 RIKEN Preventive Medicine and Diagnosis Innovation Program, Yokohama, Japan
7 Tokyo Metropolitan Institute of Medical Science, Research Center for Genome & Medical Sciences, Tokyo, Japan
8 TLaboratory for Advanced Genomics Circuit, RIKEN Center for Integrative Medical Sciences, Kanagawa, Japan
9Contributed equally to this work
*Corresponding authors:
Hideya Kawaji, PhD
E-mail: kawaji-hd@igakuken.or.jp
2-1-6 Kamikitazawa, Setagaya-ku, Tokyo, 156-8506, Japan
Yasuhisa Terao, MD, PhD
E-mail: yterao@juntendo.ac.jp
2-1-1 Hongo, Bunkyo, Tokyo 113-8421, Japan

## Slide 2
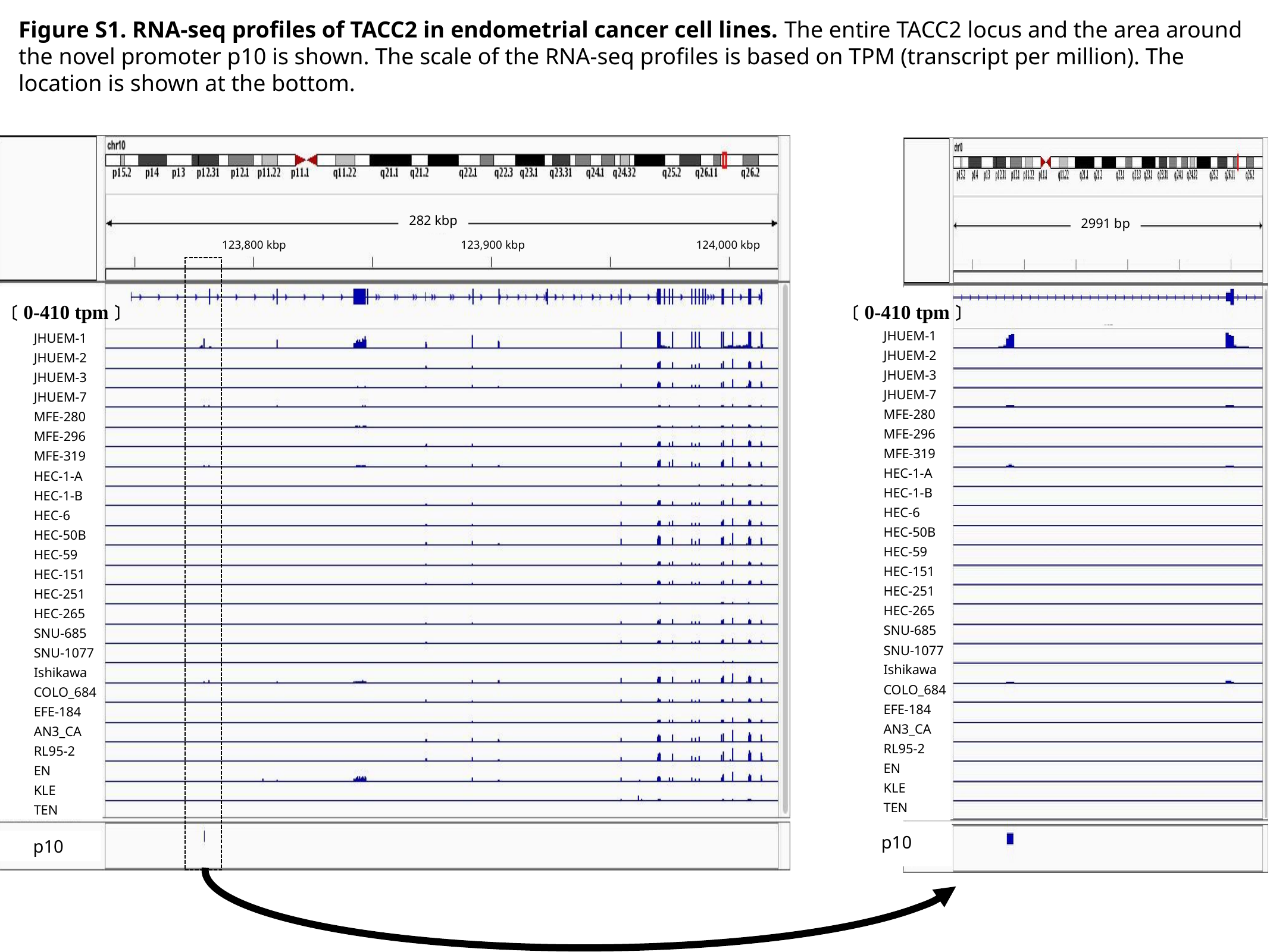

Figure S1. RNA-seq profiles of TACC2 in endometrial cancer cell lines. The entire TACC2 locus and the area around the novel promoter p10 is shown. The scale of the RNA-seq profiles is based on TPM (transcript per million). The location is shown at the bottom.
282 kbp
2991 bp
123,800 kbp
123,900 kbp
124,000 kbp
〔0-410 tpm〕
〔0-410 tpm〕
JHUEM-1
JHUEM-2
JHUEM-3
JHUEM-7
MFE-280
MFE-296
MFE-319
HEC-1-A
HEC-1-B
HEC-6
HEC-50B
HEC-59
HEC-151
HEC-251
HEC-265
SNU-685
SNU-1077
Ishikawa
COLO_684
EFE-184
AN3_CA
RL95-2
EN
KLE
TEN
JHUEM-1
JHUEM-2
JHUEM-3
JHUEM-7
MFE-280
MFE-296
MFE-319
HEC-1-A
HEC-1-B
HEC-6
HEC-50B
HEC-59
HEC-151
HEC-251
HEC-265
SNU-685
SNU-1077
Ishikawa
COLO_684
EFE-184
AN3_CA
RL95-2
EN
KLE
TEN
p10
p10

## Slide 3
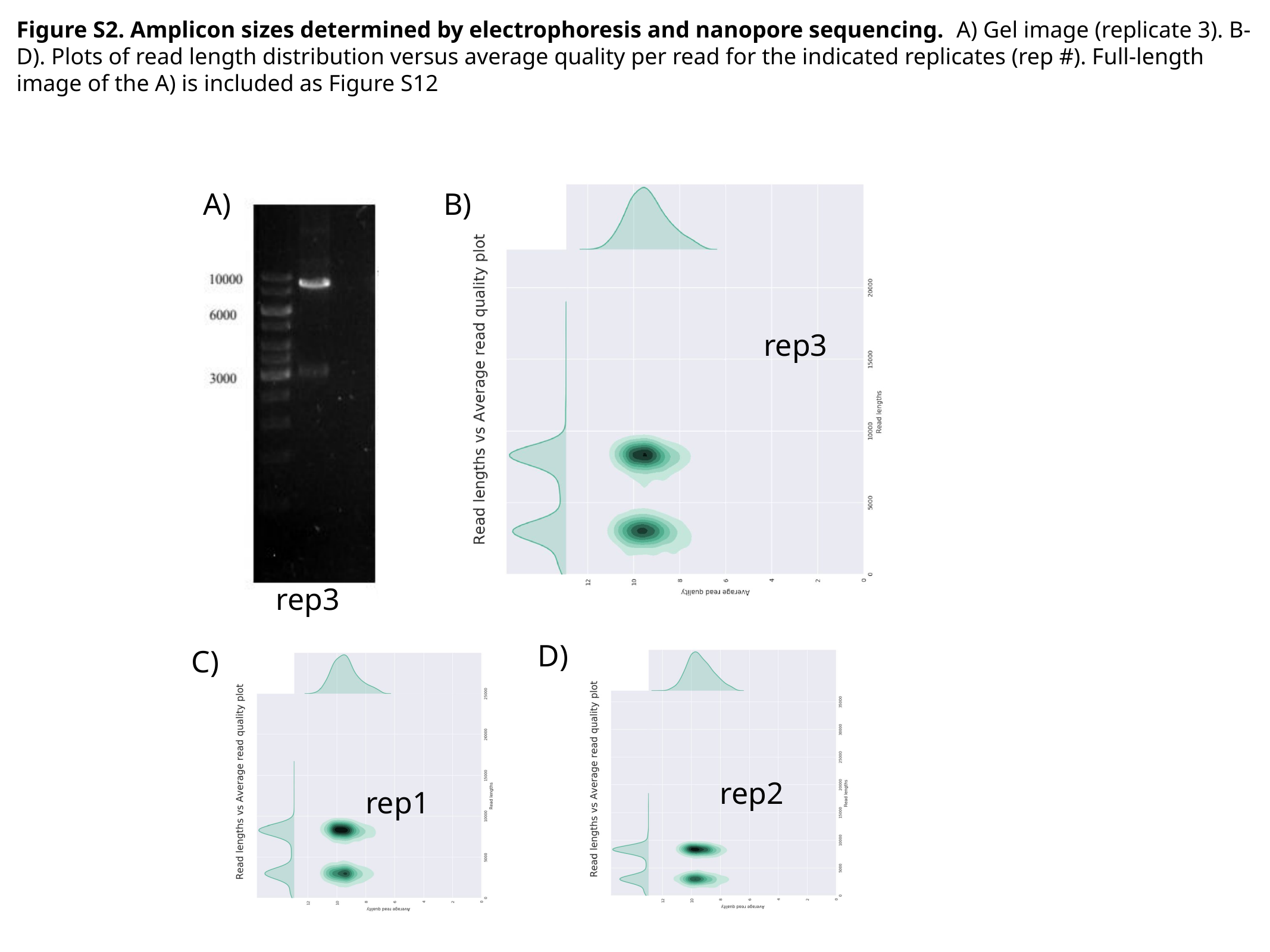

Figure S2. Amplicon sizes determined by electrophoresis and nanopore sequencing. A) Gel image (replicate 3). B-D). Plots of read length distribution versus average quality per read for the indicated replicates (rep #). Full-length image of the A) is included as Figure S12
A)
B)
rep3
rep3
D)
C)
rep2
rep1

## Slide 4
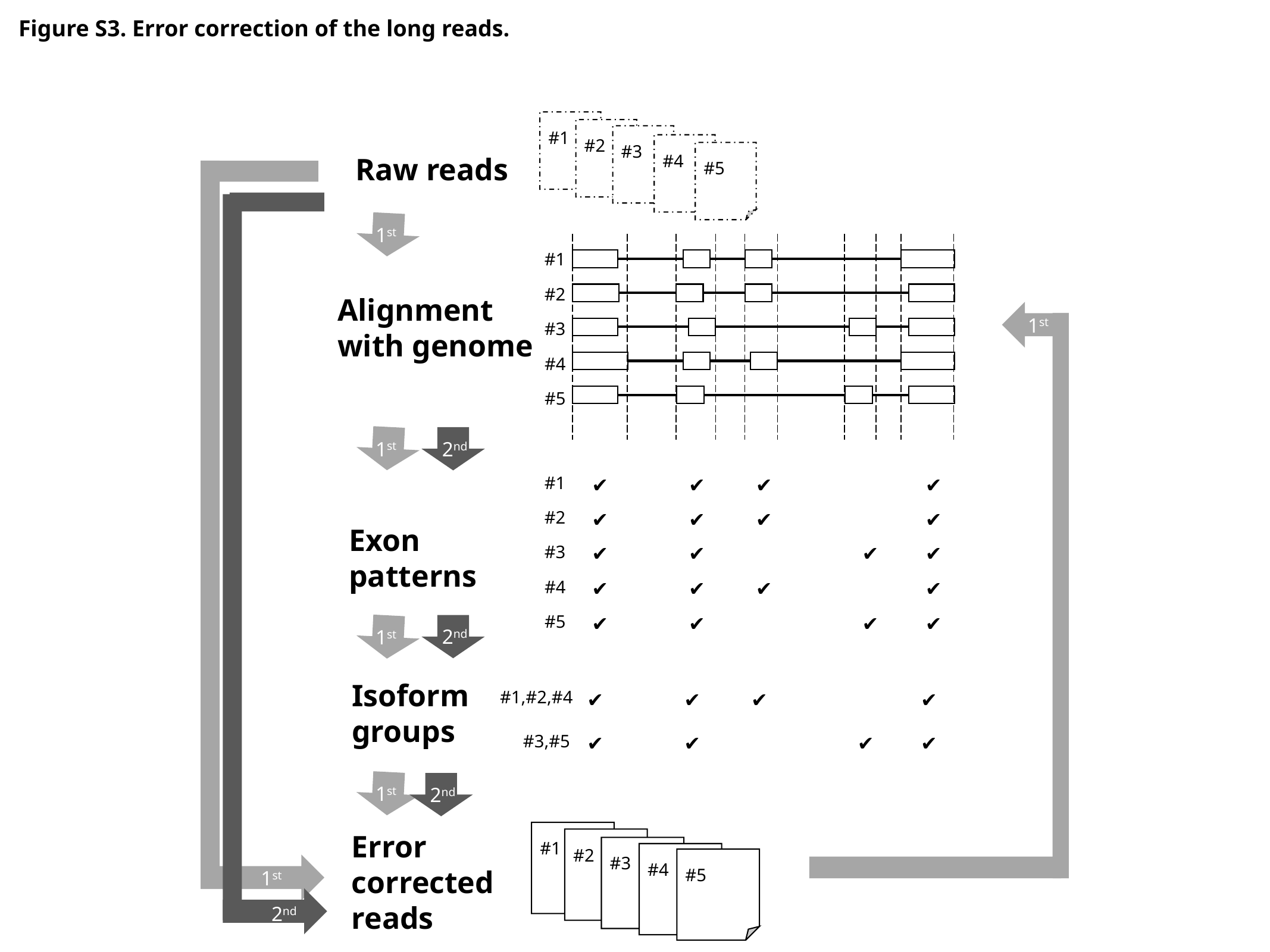

Figure S3. Error correction of the long reads.
#1
#2
#3
#4
#5
Raw reads
1st
#1
#2
Alignment
with genome
1st
#3
#4
#5
1st
2nd
#1
✔️
✔️
✔️
✔️
#2
✔️
✔️
✔️
✔️
Exon
patterns
✔️
✔️
✔️
✔️
#3
#4
✔️
✔️
✔️
✔️
#5
✔️
✔️
✔️
✔️
2nd
1st
Isoform
groups
#1,#2,#4
✔️
✔️
✔️
✔️
#3,#5
✔️
✔️
✔️
✔️
1st
2nd
Error
corrected
reads
#1
#2
#3
#4
#5
1st
2nd

## Slide 5
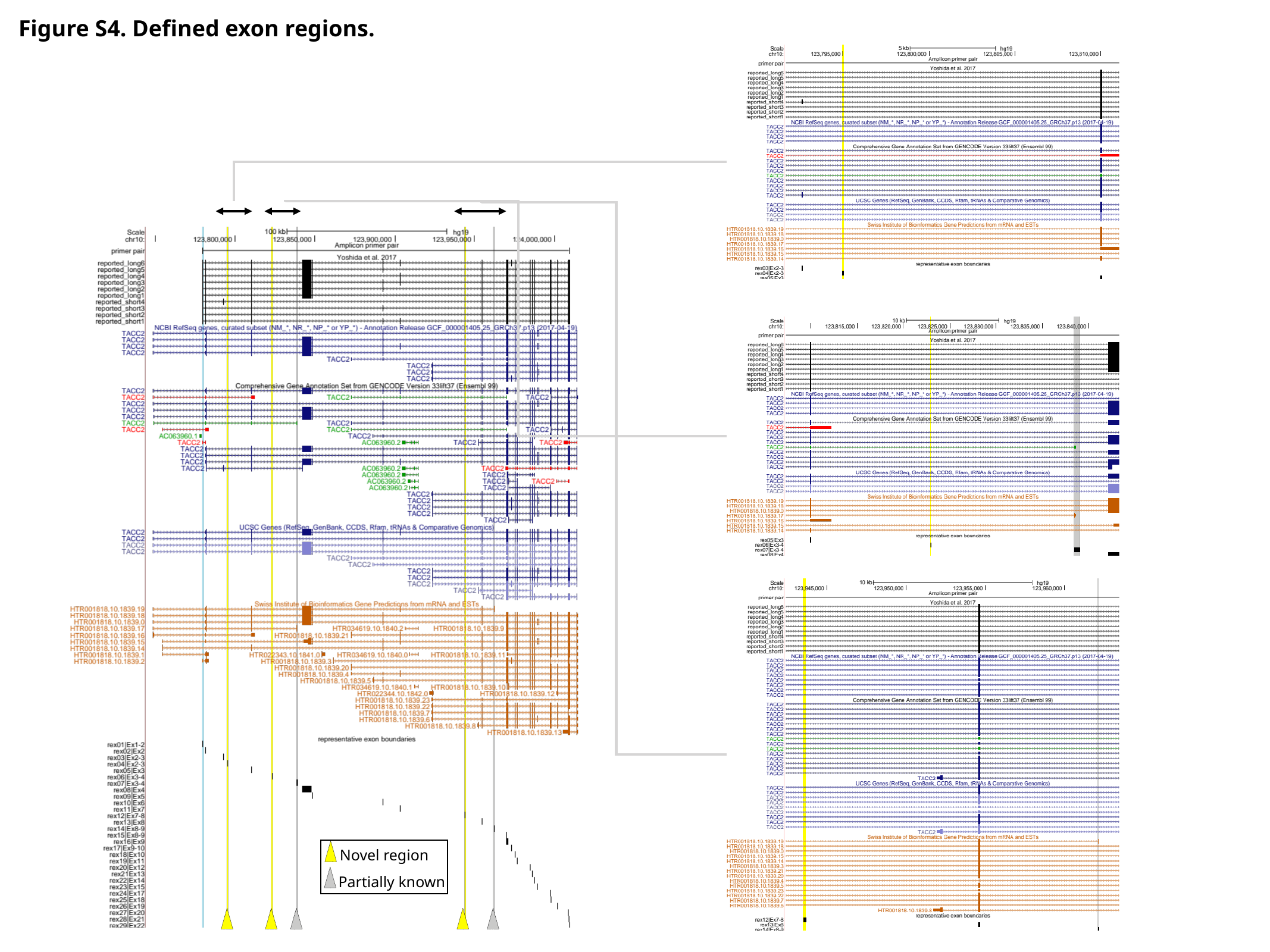

Figure S4. Defined exon regions.
Novel region
Partially known

## Slide 6
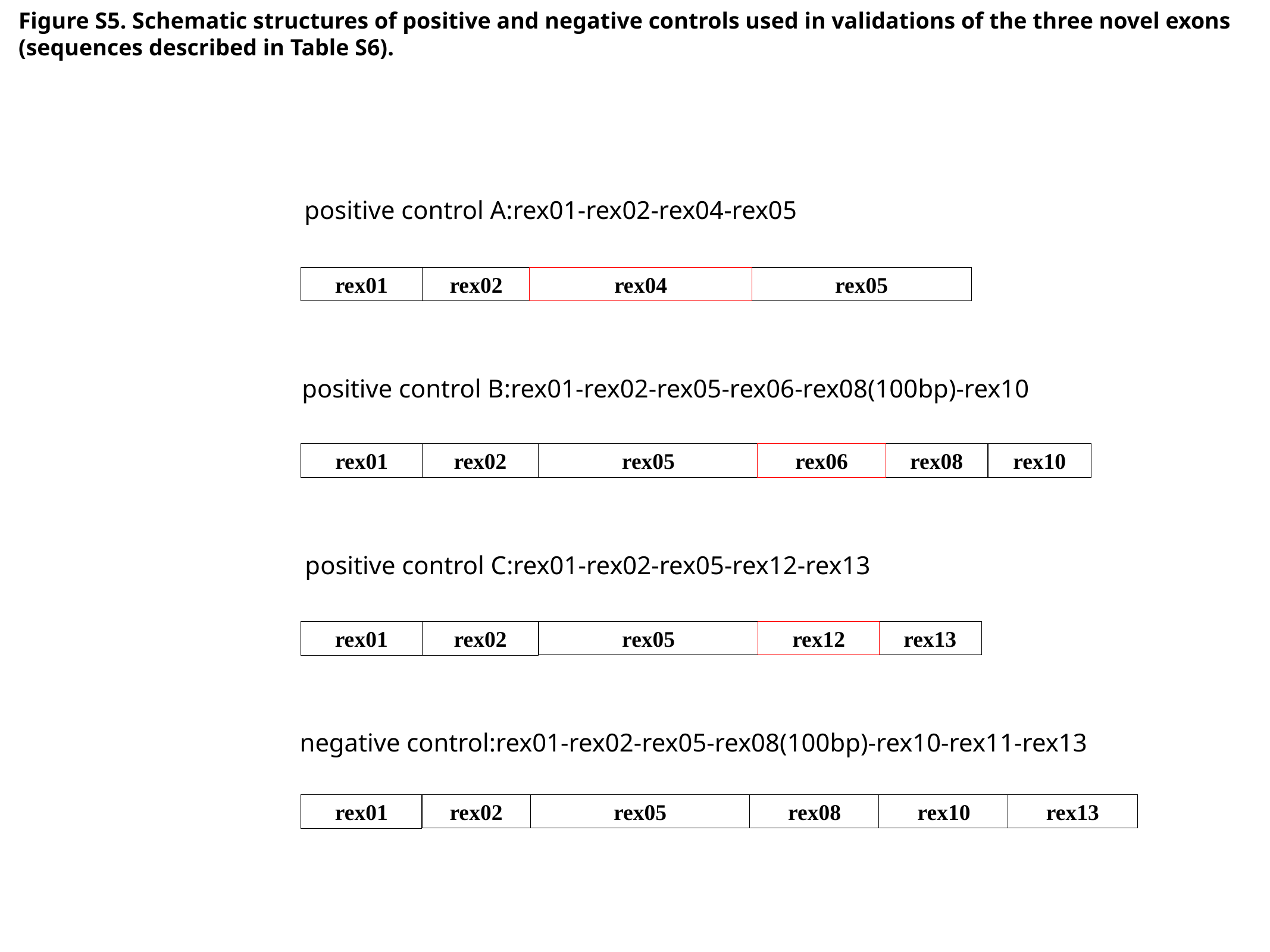

Figure S5. Schematic structures of positive and negative controls used in validations of the three novel exons (sequences described in Table S6).
positive control A:rex01-rex02-rex04-rex05
rex02
rex04
rex05
rex01
positive control B:rex01-rex02-rex05-rex06-rex08(100bp)-rex10
rex05
rex06
rex08
rex10
rex01
rex02
positive control C:rex01-rex02-rex05-rex12-rex13
rex05
rex12
rex13
rex01
rex02
negative control:rex01-rex02-rex05-rex08(100bp)-rex10-rex11-rex13
rex02
rex05
rex08
rex10
rex13
rex01

## Slide 7
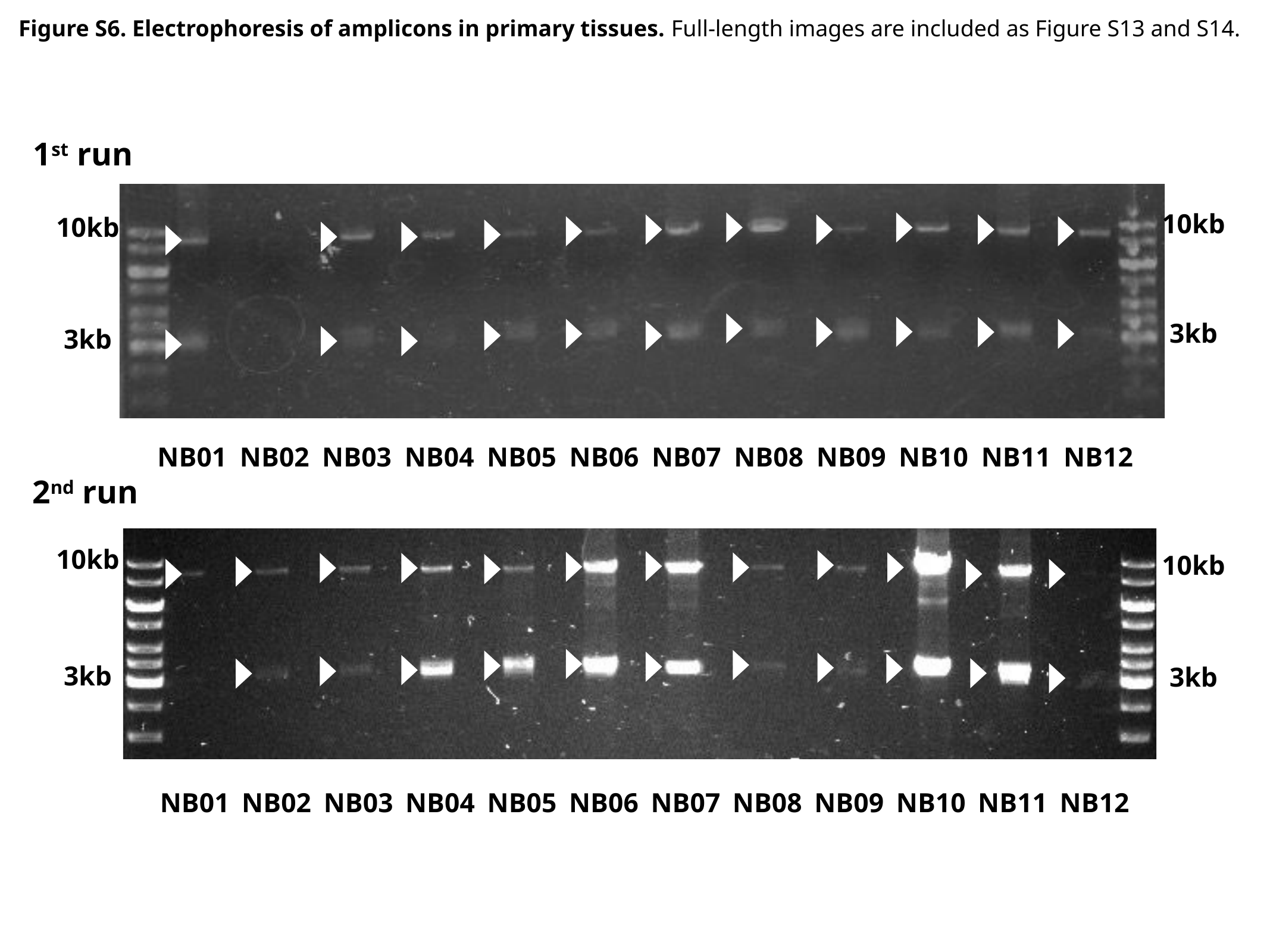

Figure S6. Electrophoresis of amplicons in primary tissues. Full-length images are included as Figure S13 and S14.
1st run
10kb
3kb
10kb
3kb
N2:5’ primer C 108b
NB01
NB02
NB03
NB04
NB05
NB06
NB07
NB08
NB09
NB10
NB11
NB12
2nd run
10kb
3kb
10kb
3kb
NB01
NB02
NB03
NB04
NB05
NB06
NB07
NB08
NB09
NB10
NB11
NB12

## Slide 8
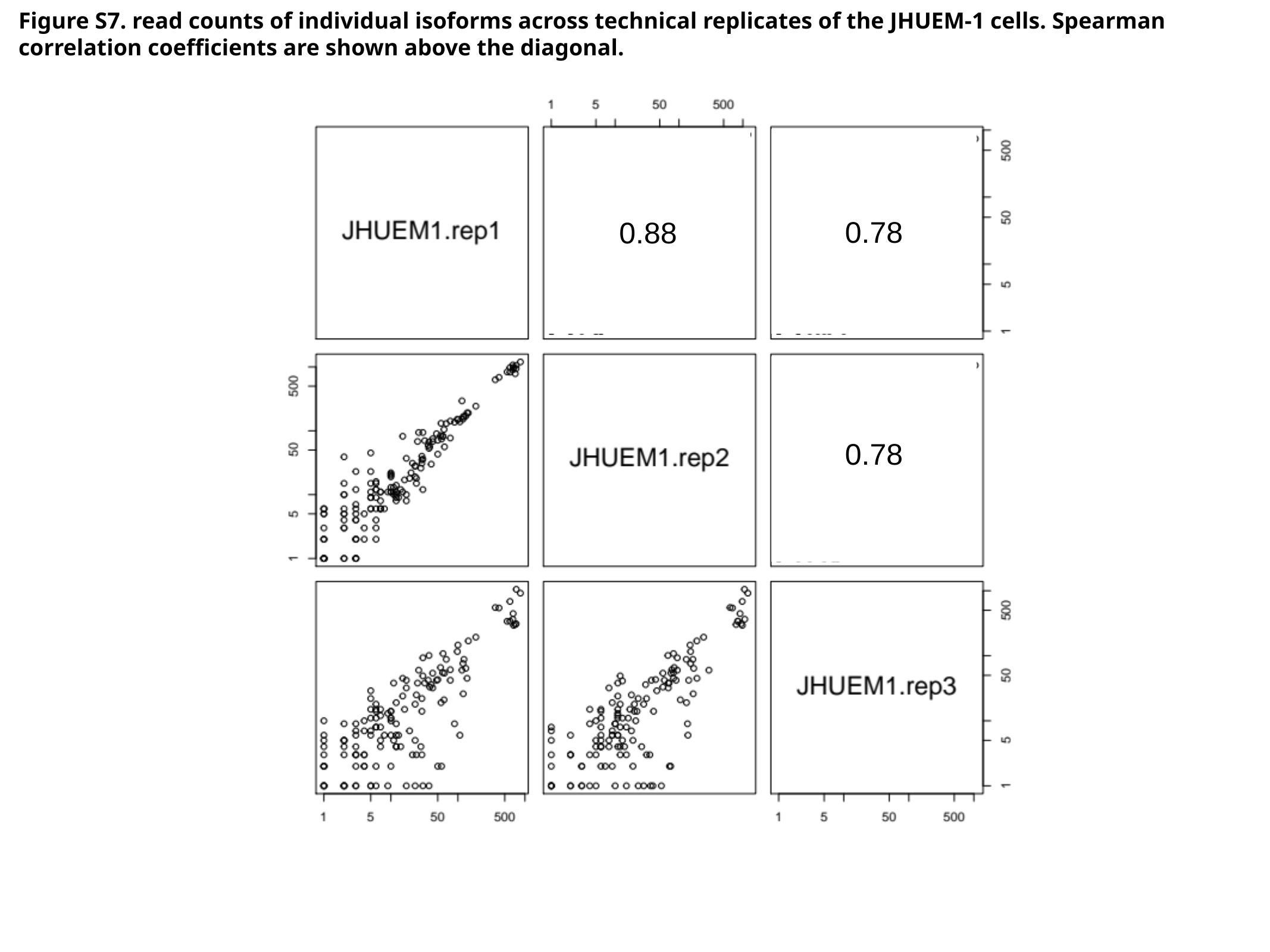

Figure S7. read counts of individual isoforms across technical replicates of the JHUEM-1 cells. Spearman correlation coefficients are shown above the diagonal.
0.78
0.88
0.78

## Slide 9
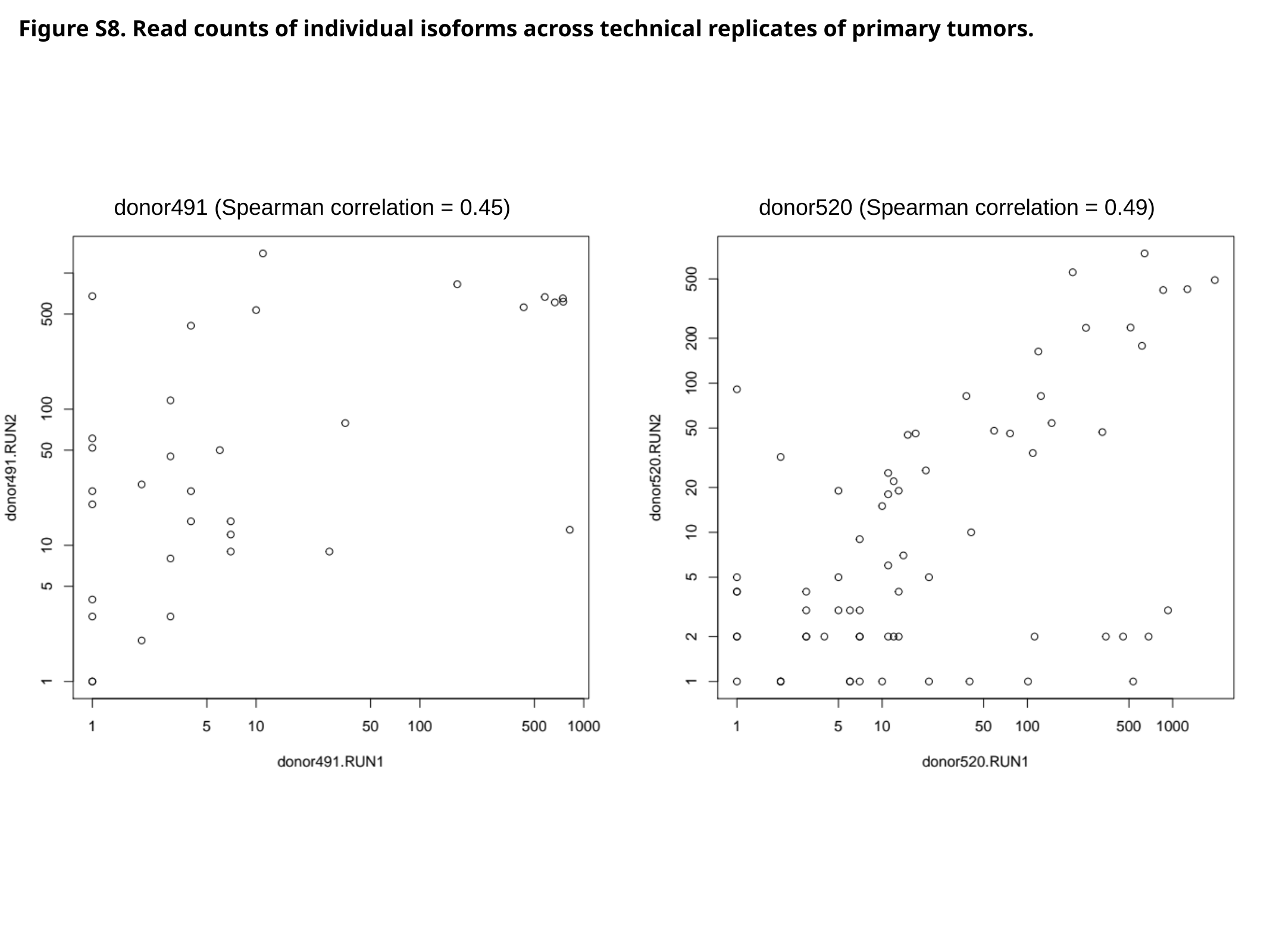

Figure S8. Read counts of individual isoforms across technical replicates of primary tumors.
donor491 (Spearman correlation = 0.45)
donor520 (Spearman correlation = 0.49)

## Slide 10
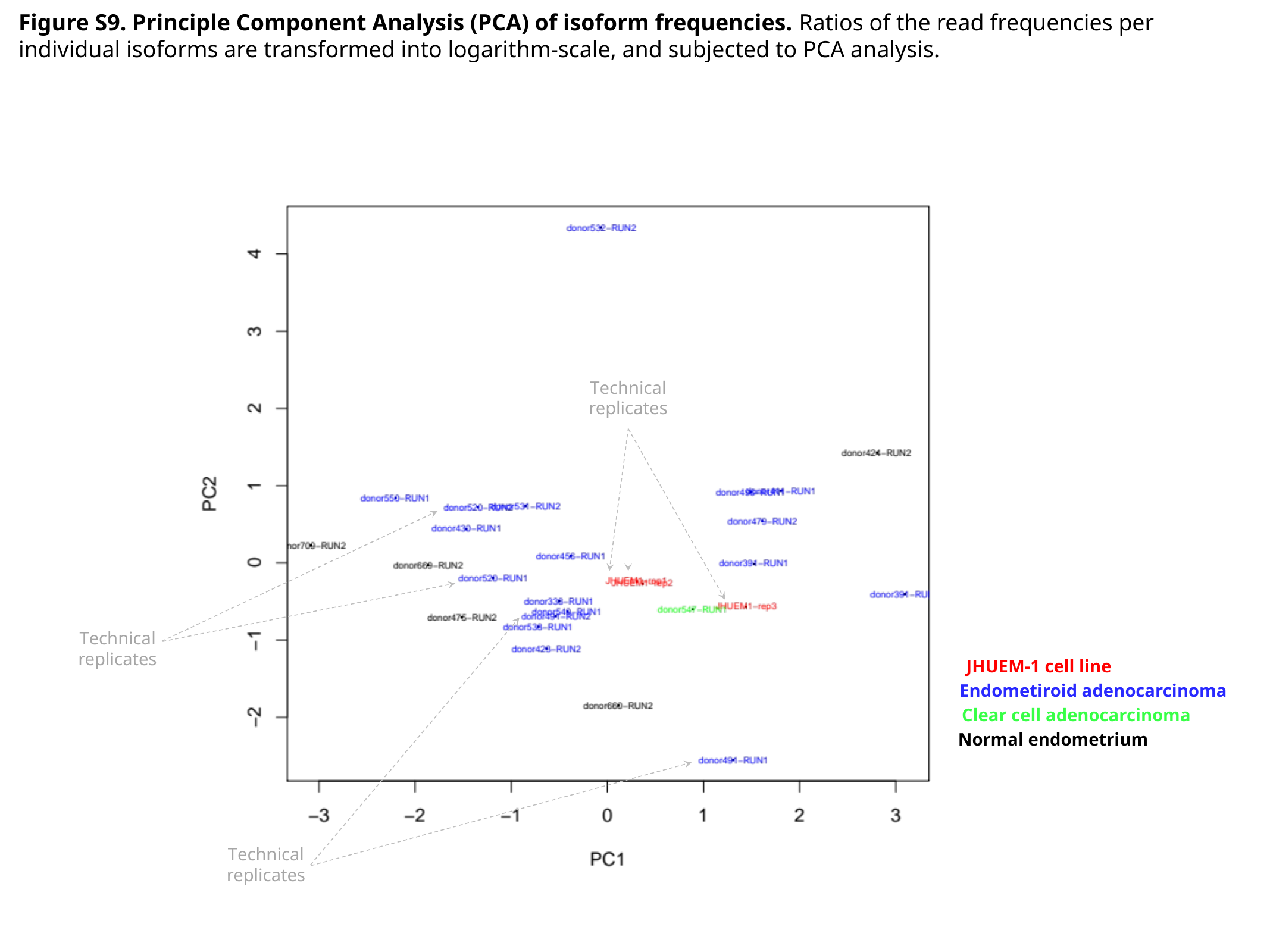

Figure S9. Principle Component Analysis (PCA) of isoform frequencies. Ratios of the read frequencies per individual isoforms are transformed into logarithm-scale, and subjected to PCA analysis.
Technical
replicates
Technical
replicates
JHUEM-1 cell line
Endometiroid adenocarcinoma
Clear cell adenocarcinoma
Normal endometrium
Technical
replicates

## Slide 11
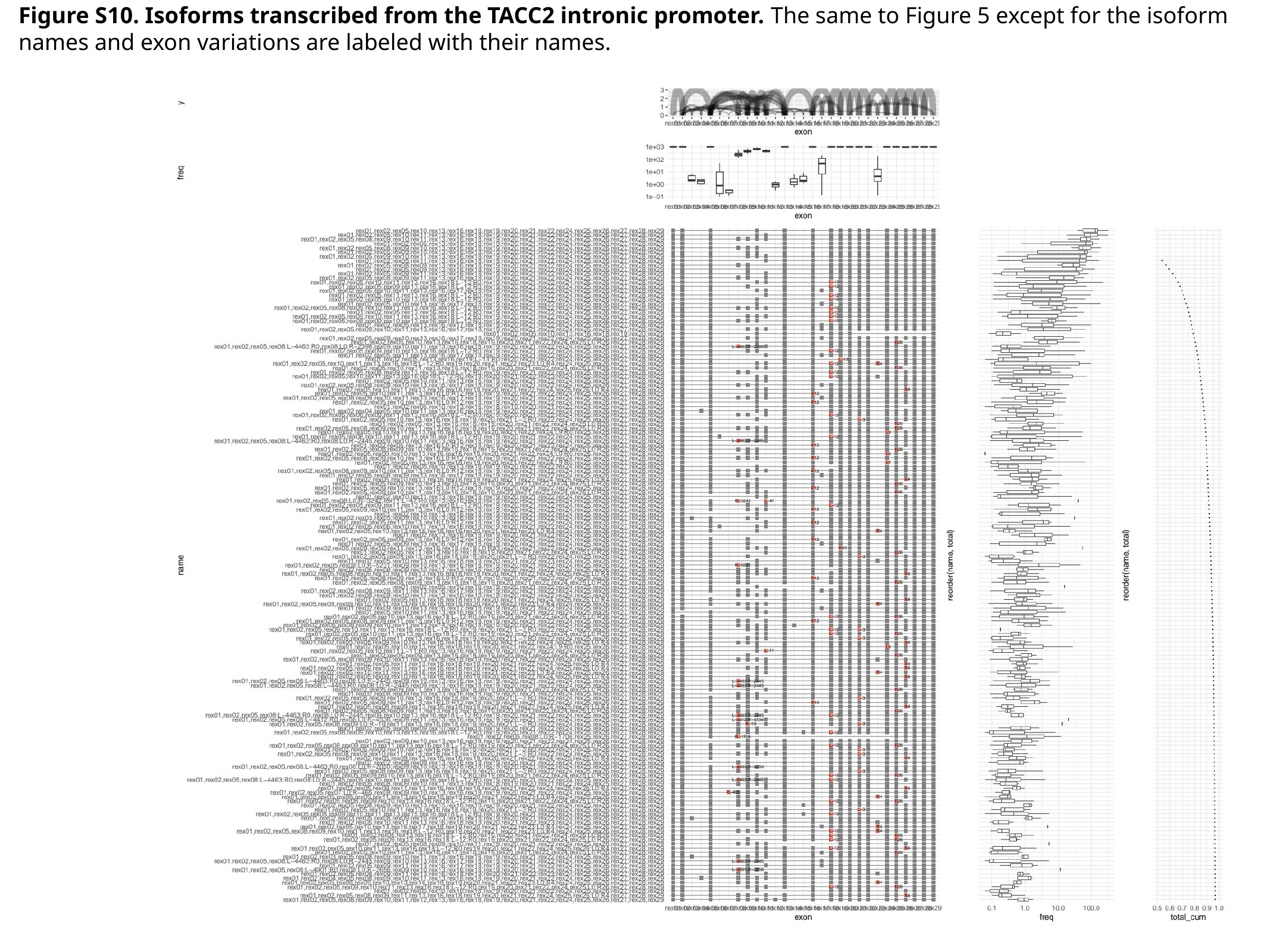

Figure S10. Isoforms transcribed from the TACC2 intronic promoter. The same to Figure 5 except for the isoform names and exon variations are labeled with their names.

## Slide 12
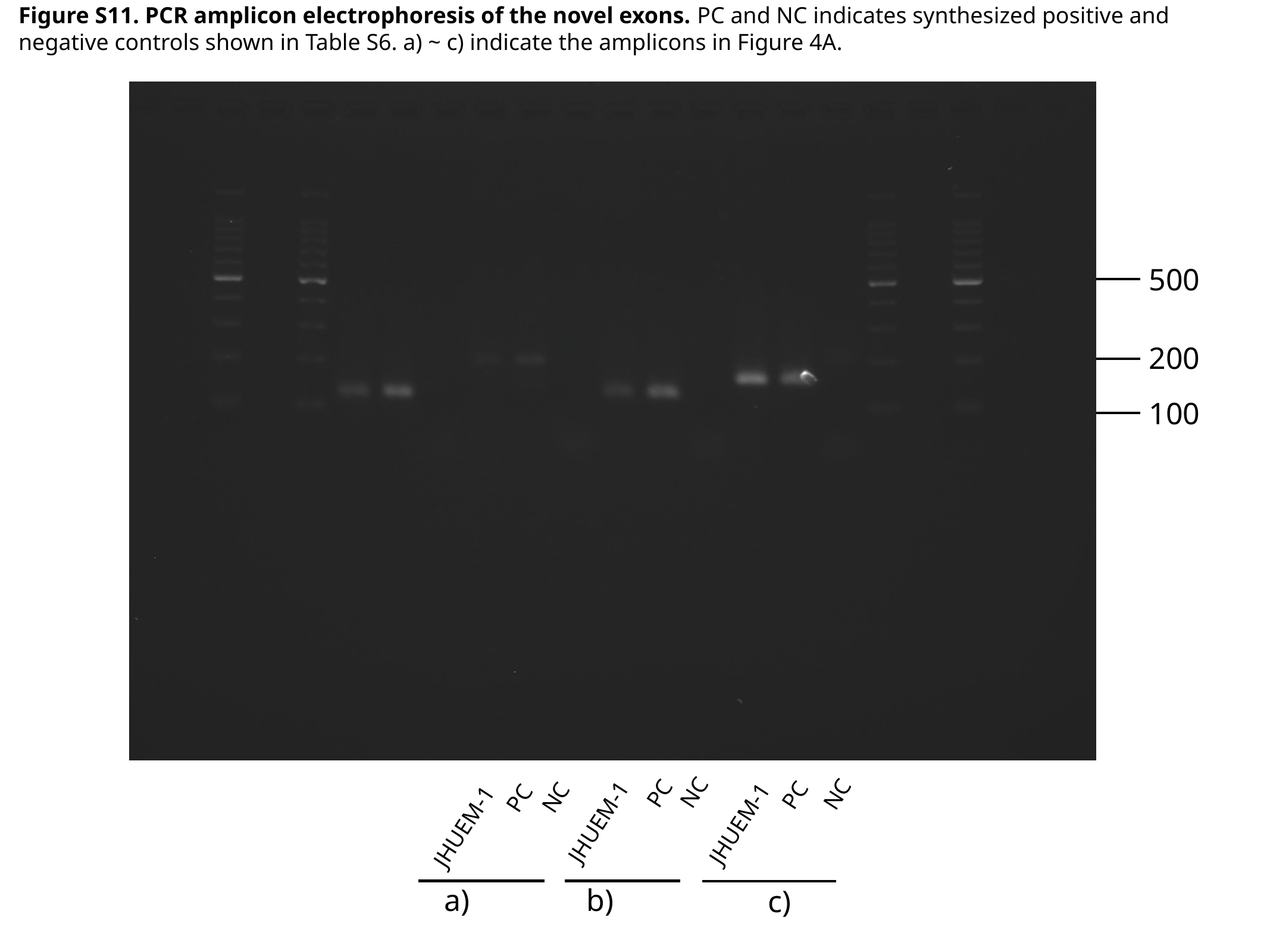

Figure S11. PCR amplicon electrophoresis of the novel exons. PC and NC indicates synthesized positive and negative controls shown in Table S6. a) ~ c) indicate the amplicons in Figure 4A.
500
200
100
NC
PC
NC
PC
NC
PC
JHUEM-1
JHUEM-1
JHUEM-1
a)
b)
c)

## Slide 13
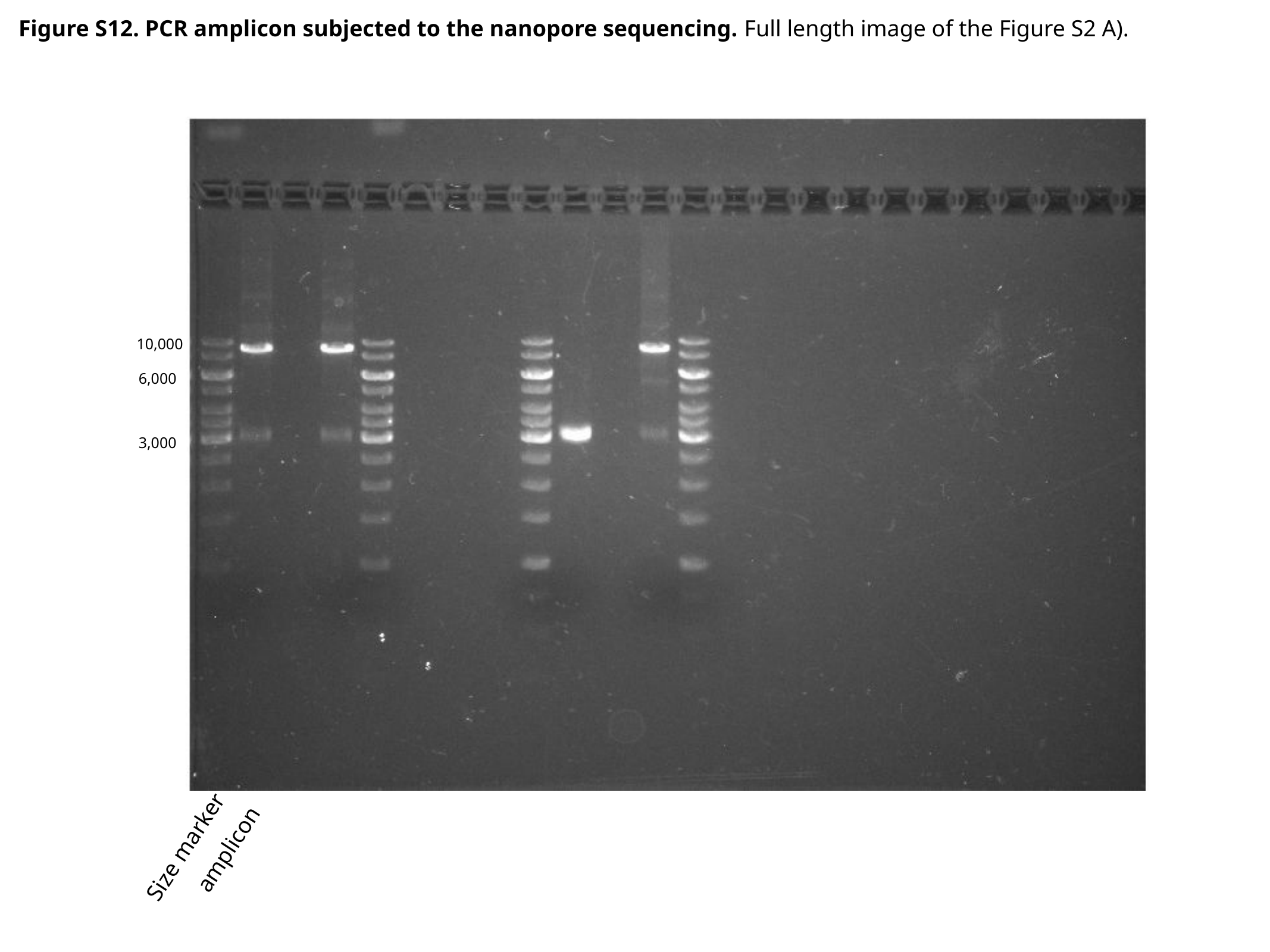

Figure S12. PCR amplicon subjected to the nanopore sequencing. Full length image of the Figure S2 A).
10,000
6,000
3,000
Size marker
amplicon

## Slide 14
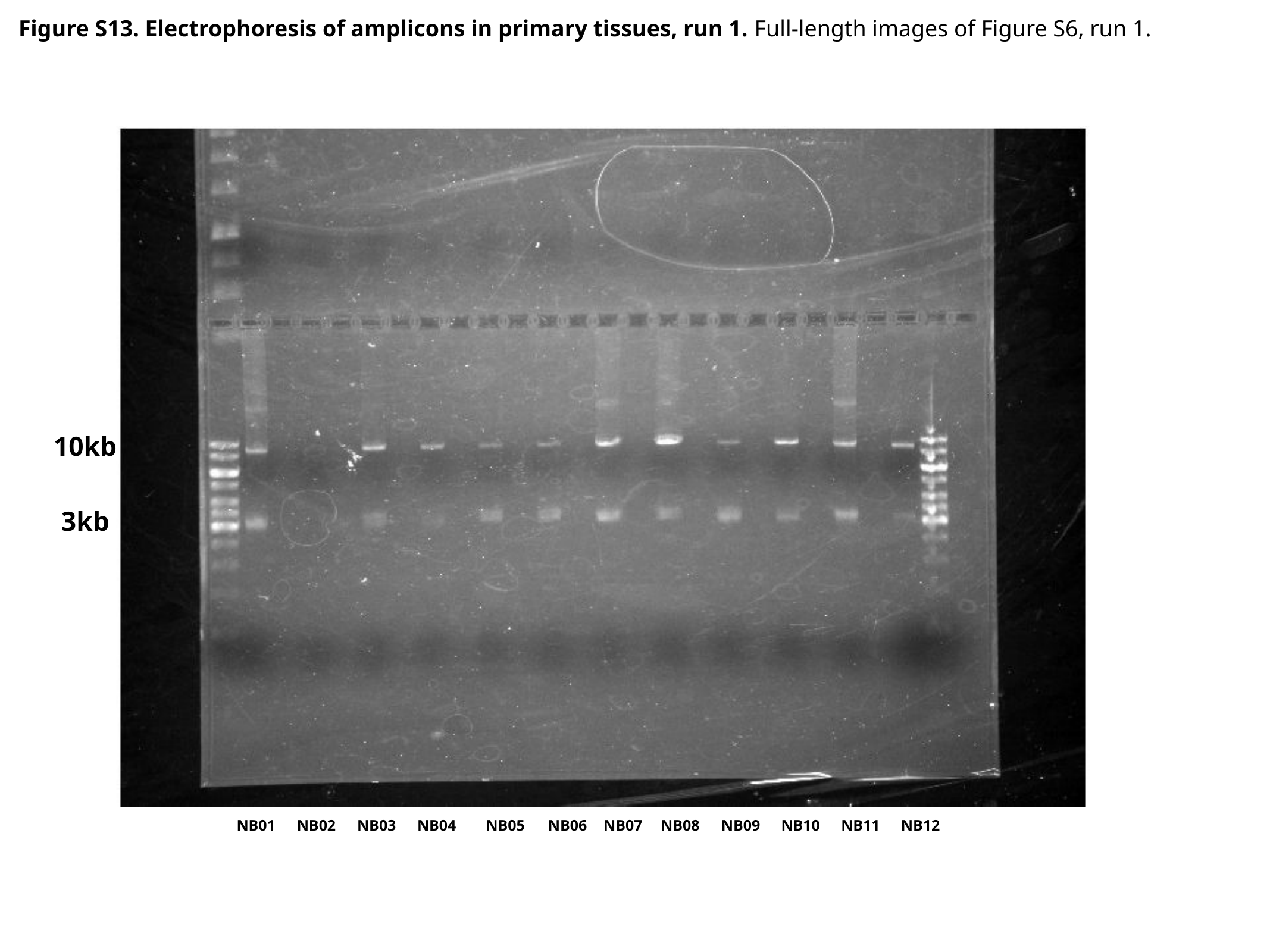

Figure S13. Electrophoresis of amplicons in primary tissues, run 1. Full-length images of Figure S6, run 1.
10kb
3kb
NB01
NB02
NB03
NB04
NB05
NB06
NB07
NB08
NB09
NB10
NB11
NB12

## Slide 15
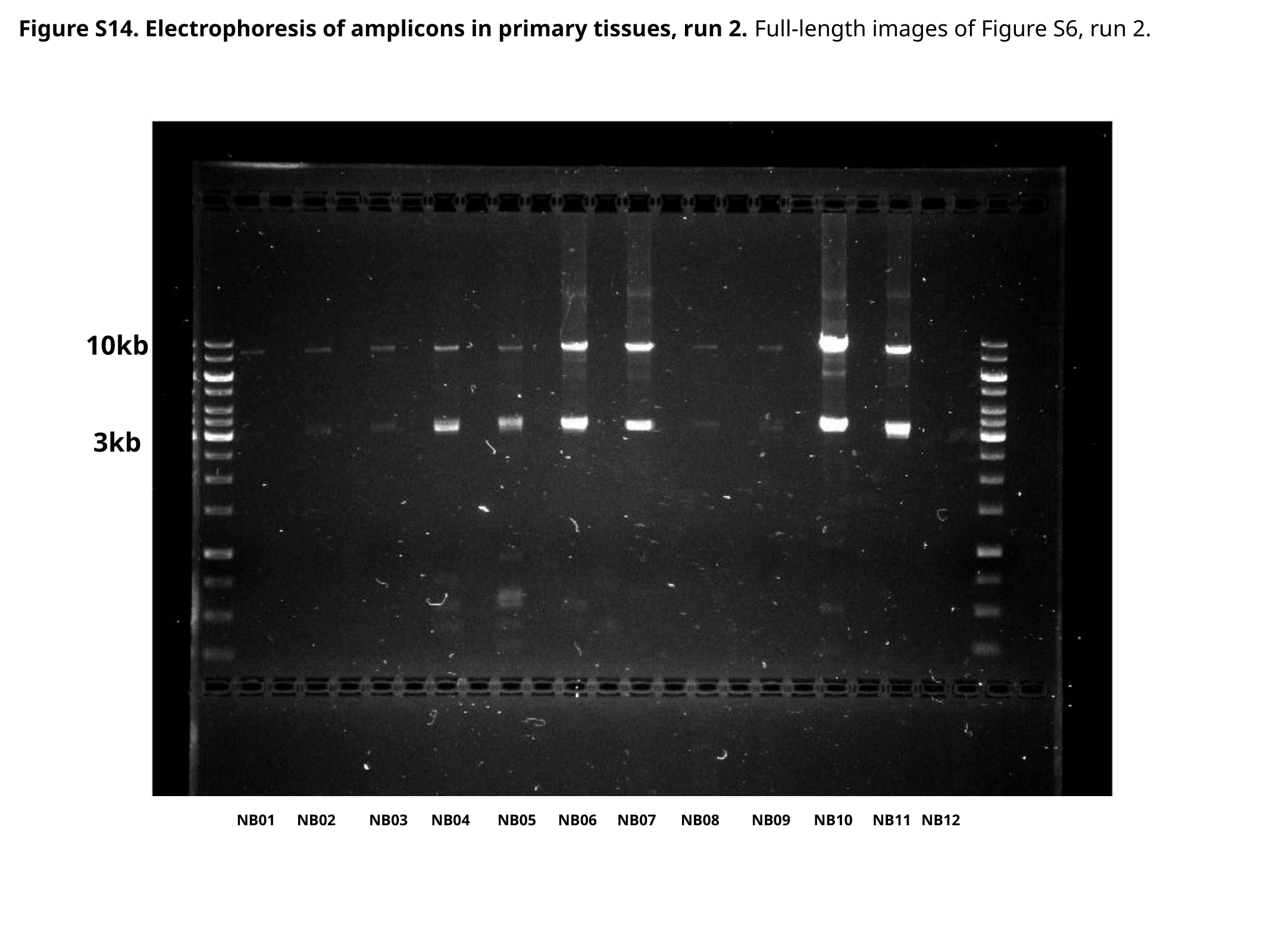

Figure S14. Electrophoresis of amplicons in primary tissues, run 2. Full-length images of Figure S6, run 2.
10kb
3kb
NB01
NB02
NB03
NB04
NB05
NB06
NB07
NB08
NB09
NB10
NB11
NB12
